# New Triazole-Isoxazole Hybrids as Antibacterial Agents: Design, Synthesis, Characterization, In Vitro, and In Silico Studies

**DOI:** 10.3390/molecules29112510

**Published:** 2024-05-26

**Authors:** Rachid Bouzammit, Salim Belchkar, Mohamed El fadili, Youssra Kanzouai, Somdutt Mujwar, Mohammed M. Alanazi, Mohammed Chalkha, Asmae Nakkabi, Mohamed Bakhouch, Emese Gal, Luiza Ioana Gaina, Ghali al houari

**Affiliations:** 1 Engineering Laboratory of Organometallic and Molecular Materials and Environment, Faculty of Sciences Dhar El Mahraz, University Sidi Mohamed Ben Abdellah, P.O. Box 1796, Atlas, Fez 30000, Morocco; youssra.kanzouai@usmba.ac.ma (Y.K.); mohammed.chalkha1@usmba.ac.ma (M.C.); asmaenakkabi@yahoo.fr (A.N.); ghalialhouari@gmail.com (G.a.h.); 2 Laboratory of Epidemiology and Research in Health Sciences, Faculty of Medicine, Pharmacy and Dentistry, University Sidi Mohammed Ben Abdellah, Fez 30070, Morocco; salim.belchkar@usmba.ac.ma; 3 LIMAS Laboratory, Chemistry Department, Faculty of Sciences Dhar El Mahraz, Sidi Mohamed Ben Abdellah University, P.O. Box 1796, Atlas, Fez 30000, Morocco; mohamed.elfadili@usmba.ac.ma; 4 Chitkara College of Pharmacy, Chitkara University, Rajpura 140401, Punjab, India; somdutt.mujwar@chitkara.edu.in; 5 Department of Pharmaceutical Chemistry, College of Pharmacy, King Saud University, Riyadh 11451, Saudi Arabia; mmalanazi@ksu.edu.sa; 6 Laboratory of Materials Engineering for the Environment and Natural Ressources, Faculty of Sciences and Techniques, University of Moulay Ismail of Meknès, B.P 509, Boutalamine, Errachidia 52000, Morocco; 7 Bioorganic Chemistry Team, Faculty of Science, Chouaïb Doukkali University, P.O. Box 24, El Jadida 24000, Morocco; bakhouch.m@ucd.ac.ma; 8 Research Center on Fundamental and Applied Heterochemistry, Faculty of Chemistry and Chemical Engineering, Babeş-Bolyai University, 11 Arany Janos Str., RO-400028 Cluj-Napoca, Romania; emese.gal@ubbcluj.ro (E.G.); gaina.ioana.luiza@gmail.com (L.I.G.)

**Keywords:** synthesis, triazole, isoxazole, antibacterial activity, molecular docking, dynamic molecular, ADMET properties

## Abstract

Novel isoxazole–triazole conjugates have been efficiently synthesized using 3-formylchromone as starting material according to a multi-step synthetic approach. The structures of the target conjugates and intermediate products were characterized by standard spectroscopic techniques (^1^H NMR and ^13^C NMR) and confirmed by mass spectrometry (MS). The all-synthesized compounds were screened for their antibacterial activity against three ATCC reference strains, namely *Staphylococcus aureus* ATCC 25923, *Staphylococcus aureus* ATCC BAA-44, and *Escherichia coli* ATCC 25922 as well as one strain isolated from the hospital environment *Pseudomonas aeruginosa*. The findings indicate that conjugate 7b exhibits a stronger antibacterial response against the tested *Escherichia coli* ATCC 25922 and *Pseudomonas aeruginosa* pathogenic strains compared to the standard antibiotics. Furthermore, hybrid compound **7b** proved to have a bactericidal action on the *Escherichia coli* ATCC 25922 strain, as evidenced by the results of the MBC determination. Moreover, the ADMET pharmacokinetic characteristics revealed a favorable profile for the examined compound, as well as a good level of oral bioavailability. Molecular docking and molecular dynamics simulations were performed to explore the inhibition mechanism and binding energies of conjugate **7b** with the proteins of *Escherichia coli* and *Pseudomonas aeruginosa* bacterial strains. The in silico results corroborated the data observed in the in vitro evaluation for compound **7b**.

## 1. Introduction 

Heterocyclic structures containing nitrogen and oxygen often serve as the basis in various classes of natural compounds, as well as fundamental scaffolds for many drugs [1]. They are also found in numerous herbicides, fungicides, insecticides, and corrosion protection applications [2,3,4]. Owing to their significant importance, chemists continuously strive to synthesize these structures selectively and efficiently.

**Figure 1:**
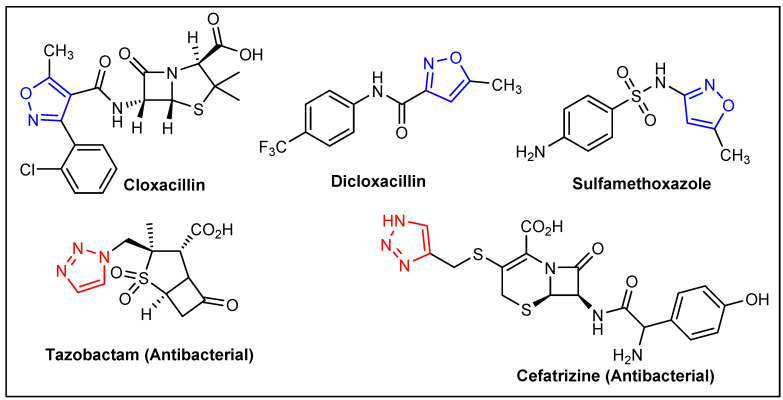
Examples of biologically active compounds incorporating a triazole or isoxazole nucleus.

Among heterocyclic compounds, triazoles have received considerable attention over the last decade due to their distinct chemical and structural characteristics, as well as their wide-ranging applications in medicinal chemistry [5,6,7,8]. They are versatile compounds that have demonstrated efficacy in combating a wide range of infectious diseases, including bacterial [9], fungal [10], protozoal [11], and even viral infections [12]. Additionally, the triazole nucleus is also found in the structure of several drugs available in the pharmaceutical market, such as tazobactam, cefatrizine, etc. (Figure 1). This makes them interesting candidates for the development of new drugs and therapies. Similarly, isoxazoles are part of the chemical family of heterocyclic compounds, which have attracted significant interest both synthetically and biologically due to their numerous potential applications in various fields [13,14]. These nuclei are often used as synthetic intermediates in the preparation of many active pharmaceutical ingredients (Figure 1) [15]. Indeed, they are associated with a variety of diverse pharmacological properties, including antiviral [16], antitumoral [17], antimicrobial [18], antiproliferative [19], anti-inflammatory [20], anticancer [21,22], anti-Alzheimer’s [21], and many others [23].

Recently, a new strategy involving the combination of two or more biologically active structural motifs has emerged as promising for generating a new class of therapeutic agents. This approach has proven highly effective against the growth of bacteria known for their antibiotic resistance. Within this framework, the combination of biologically interesting core structural motifs [24,25], such as the triazole, with a further heterocyclic nucleus holds therapeutic properties in a variety of fields, including combating microbial pathogens [26]. Particularly, diversely substituted triazole/isoxazole conjugates have shown enhanced biological activities [27,28,29].

On the other hand, chromones represent an intriguing class of heterocyclic entities rich in pharmacological potential and are found in numerous plants [30,31]. These systems are commonly applied in organic chemistry as intermediate compounds for the synthesis of new therapeutic agents, including antitumor agents [32] antimicrobials [33,34], fungicides [35,36], insecticides [37,38] and antivirals [39].

Given the biological significance of heterocycles, and in continuation of our research efforts focused on the synthesis and development of new bioactive heterocycles [40,41,42], the study undertaken in the present work aims at the synthesis and structural identification of a novel range of heterocyclic molecules possessing a triazole and isoxazole as the basic structure, derived from 3-formylchromone using N-alkylation and 1,3-DC reactions. Furthermore, the synthesized heterocyclic compounds have been evaluated for their antibacterial activities through both in vitro and in silico studies.

## 2. Results and Discussion

### 2.1. Synthesis and Characterization

#### 2.1.1. Reaction of Benzyl Azide and 3-Formylchromone

**Scheme 1:**
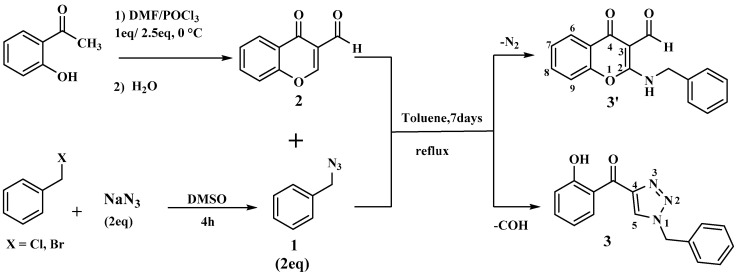
The reaction between benzyl azide and 3-formylchromone yielded two products: 1-benzyl-4-(2-hydroxybenzoyl)-1,2,3-triazole **3** and 2-aminobenzyl-3-formylchromone **3′**, synthesized from the reaction between benzyl azide and 3-formylchromone.

In order to synthesize new tricyclic compounds bearing the triazoline moiety by performing 1,3-DC reactions of benzyl azide onto the endocyclic double bound of the α,β-unsaturated carbonyl system, we chose to react benzyl azide **1** with 3-formylchromone **2** in toluene under basic conditions (Scheme 1). The reagents **1** and **2** were synthesized following procedures described in the literature [43,44]. The reaction resulted in the formation of two unexpected products after the consumption of the basic product. Upon separation and purification of these two products, we observed that the first product, 2-aminobenzyl-3-formylchromone **3′**, was obtained after spontaneous denitrogenation of the intermediate tetrazolic cycle formed, while the compound 1-benzyl-4-(2-hydroxybenzoyl)-1,2,3-triazole **3** was generated in an unusual way after ring-opening of the pyrone moiety. 

**Scheme 2:**
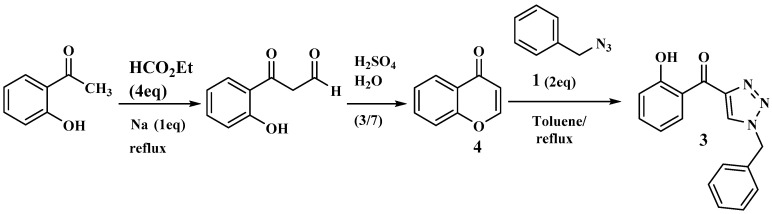
Pathway for synthesis of 1-benzyl-4-(2-hydroxybenzoyl)-1,2,3-triazole **3** from unsubstituted chromone.

**Scheme 3:**
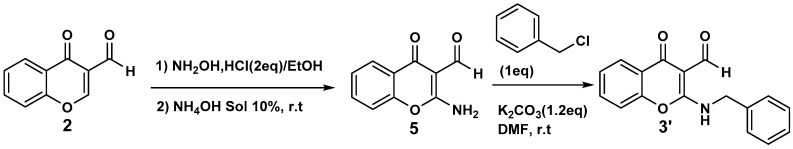
Path of the synthesis of 2-aminobenzyl-3-formylchromone **3′** from 2-amino-3-formylchromone.

In order to understand the transformations involved and to validate the proposed findings, we attempted to synthesize the two products using two different synthetic routes from those previously conducted. The first synthetic method for the compound 1-benzyl-4-(2-hydroxybenzoyl)-1,2,3-triazole **3** involves reacting benzyl azide **1** with unsubstituted chromone **4** instead of 3-formylchromone **2** (Scheme 2). The unsubstituted chromone **4** is prepared according to literature protocols from 2-hydroxyacetophenone in two steps [45]. The reaction led to the generation of the same product as previously obtained: 1-benzyl-4-(2-hydroxybenzoyl)-1,2,3-triazole **3**. As for the compound 2-aminobenzyl-3-formylchromone **3′**, a second synthetic approach was adopted starting from 3-formylchromone 2 (Scheme 3). This strategy involves first introducing a primary amine at position 2 of chromone **2** to transform it into an intermediate **5**, 2-amino-3-formylchromone, and then N-alkylating the formed intermediate with benzyl chloride. The reaction also led to the formation of the desired product, 2-aminobenzyl-3-formylchromone **3′**.

The structures of the newly synthesized compounds 1-benzyl-4-(2-hydroxybenzoyl)-1,2,3-triazole **3** and 2-aminobenzyl-3-formylchromone **3′** were determined based on usual spectroscopic techniques and confirmed by MS. Indeed, in the IR spectrum of compound 3, we observed an absorption band around 3150 cm^−1^, which is characteristic of the vibration of the OH bond of the alcohol functional group. The ^1^H NMR spectrum of **3** displayed a singlet signal at 12.3 ppm attributable to the alcohol function proton (OH), and a singlet signal at 8.2 ppm corresponding to the H5 proton of the triazole ring, which is in accordance with the chemical shift outlined in the literature for the H5 proton of triazole [46]. Furthermore, a singlet signal integrating the two methylene protons CH2 adjacent to the nitrogen atom resonated at 5.63 ppm. In the ^13^C NMR spectrum of **3**, there was a signal around 188 ppm characteristic of the carbonyl carbon (C=O), a signal around 163 ppm characteristic of the aromatic carbon linked to the alcohol function (=C-OH), and a signal at 148 corresponding to the quaternary carbon C4 of the triazole ring, which is in good agreement with literature data [46]. Finally, a signal at 54 ppm corresponding to the methylene carbon CH2 adjacent to the nitrogen atom was observed. Additionally, the formation of **3′** was also confirmed using various spectroscopic methods. In the IR spectrum of **3′**, a weak absorption band at around 3300 cm^−1^ was observed, corresponding to the NH stretching vibration of the secondary amine. In the ^1^H NMR spectrum of this compound, a singlet signal at 10.95 ppm was observed for the proton of the secondary amine (N-H). A singlet signal at 10.22 ppm was observed corresponding to the aldehyde proton (CH=O), and finally, a doublet resonating at 4.79 ppm attributable to the protons of the CH2 group adjacent to the nitrogen atom of the secondary amine was observed. Furthermore, the ^13^C NMR spectrum showed, in addition to characteristic signals for aromatic carbons, the presence of a signal at 189.62 ppm corresponding to the carbon of the aldehyde function (CH=O) and a signal at 175.77 ppm corresponding to the carbon of the ketone function (C=O). The two signals located at 164.35 ppm and 99.52 ppm are attributable to the quaternary carbons, successively C2 and C3 of the pyran ring, and the methylene carbon CH2 bonded to the nitrogen atom resonated at 44.95 ppm. Additionally, MS confirmed the structure and purity of the synthesized compounds (**3** and **3′**). The MS data for each synthesized product are consistent with the structures proposed and to the values obtained for the molecular ions [M − H]^+^.

#### 2.1.2. Synthesis of Molecules Hybrids

a. Synthesis of precursor **6**

**Scheme 4:**
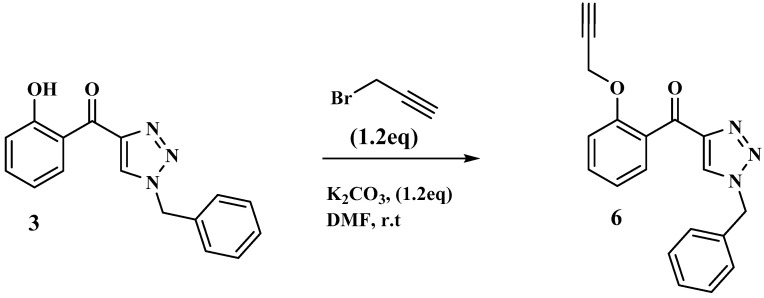
O-Alkylation reaction of 1-benzyl-4-(2-hydroxybenzoyl)-1,2,3-triazole **3**.

The newly synthesized compound 1-benzyl-4-(2-hydroxybenzoyl)-1,2,3-triazole **3** underwent O-alkylation reaction with propargyl bromide in dimethylformamide (DMF) over potassium carbonate (K_2_CO_3_) at room temperature (RT) (Scheme 4). The reaction yielded product **6** with a good yield.

The characterization of **6** was carried out based on their ^1^H, ^13^C NMR, and IR spectral data as well as by MS. In the IR spectrum of product 6, an absorption band around 3300 cm^−1^ was observed, which is characteristic of the ≡C-H bond vibration. This indicates the substitution of the secondary alcohol. The ^1^H NMR spectrum of **6** revealed the appearance of a triplet signal at 2.41 ppm attributable to the acetylenic proton (HC≡C); two successive signals at 4.63 and 5.55 ppm, the first as a doublet and the second as a singlet corresponding to the two groups (O-CH_2_) and (N-CH_2_), respectively; and a signal in the form of a characteristic singlet for the H5 proton of the triazolic ring. The disappearance of a singlet signal at 12.3 ppm, attributable to the alcohol function proton (OH) of the product 1-benzyl-4-(2-hydroxybenzoyl)-1,2,3-triazole 3, indicates the progress of the reaction towards the formation of the triazole-propargylic **6**. Additionally, the ^13^C NMR spectrum (decoupled and APT) of the same product revealed the presence of two signals at 54 and 56 ppm, attributable to the two groups (O-CH_2_) and (N-CH_2_), respectively; a signal at 75.92 ppm attributable to the acetylenic carbon (≡CH); a signal at around 148.37 ppm attributable to the quaternary carbon C4 of the triazole ring; a signal at 155.97 ppm corresponding to the quaternary benzylic carbon linked to oxygen (=C-O-); and finally a signal at 187.34 ppm corresponding to the carbonyl. Moreover, the O-propargylation of compound **3** was confirmed by mass spectrometry, which indicates the presence of a peak of the protonated molecular ion [C_19_H_15_N_3_O_2_ + H]^+^ at *m*/*z* = 318.12485 attributed to the precise mass of compound **6**.

b. Synthesis of triazole-isoxazole conjugates from precursor **6**

**Scheme 5:**
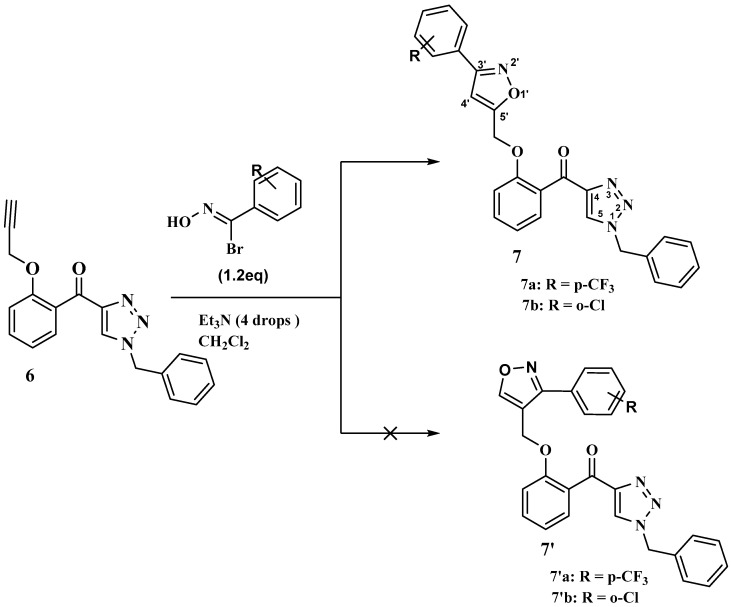
1,3-DC reaction between **6** and nitrile oxides.

Compound **6**, derived via the O-alkylation reaction with propargyl bromide, was used as a dipolarophile in the 1,3-DC reaction with 1,3-dipoles of the nitrile oxide type (Scheme 5). These reactions led to the formation of new hybrid heterocyclic systems containing in their skeleton the triazole motif coupled to the isoxazole, linked together by a 2-methoxybenzoyl linker. All the reactions undertaken proceeded correctly in all cases, yielding the desired products in a highly regioselective manner and with good yields of pure products. The structures of all-synthesized products have been established through the usual spectroscopic approaches and mass spectrometry.

The analysis of spectral data of the obtained products revealed that all 1,3-DC reactions between the propargylated triazole and nitrile oxides resulted in a single regioisomer **7**, with no trace of product **7′** (Scheme 5). This finding is fully supported by literature data on the regiochemistry of 1,3-DC reactions of nitrile oxides with similar dipolarophiles [40,47]. Indeed, on the ^1^H NMR spectra of compounds **7a**–**b**, two successive signals between 5.25 and 5.58 ppm appeared as a singlet corresponding to the two groups (O-CH_2_) and (N-CH_2_), respectively, and a singlet signal appeared around 6.64 ppm corresponding to the **H_4′_** proton of the isoxazole ring. This result is consistent with the chemical shift reported for the **H_4′_** proton in similar 3,5-disubstituted isoxazoles [48]. For the 3,4-disubstituted isoxazoles **7a′**–**b′**, the H5′ proton resonated typically around 8.5 ppm since it is deshielded by the neighboring oxygen atom [49]. The H5 proton of the triazole ring appeared around 8.06 ppm [46]. Additionally, both synthesized cycloadducts **7a**–**b** were validated by mass spectrometry, which suggested the existence of a peak of the protonated molecular ion for the two compounds, **7a** and **7b** [[C_27_H_19_F_3_N_4_O_3_ + H]^+^ and [C_26_H_19_ClN_4_O_3_ + H]^+^] at *m*/*z* = 505.14828 and *m*/*z* = 471.12320, respectively, corresponding to the exact mass of compounds **7a** and **7b**.

### 2.2. Antibacterial Assessment

#### 2.2.1. Antibiotic Susceptibility Test

The results of the Kirby–Bauer test demonstrated the sensitivity of the strains to antibiotics. The results confirm the bacterial profiles of the reference strains regarding their antibiotic sensitivity. Specifically, *Staphylococcus aureus* ATCC 25923, as indicated, exhibited sensitivity to all the tested antibiotics except for ampicillin, thereby confirming its sensitive profile. In contrast, *Staphylococcus aureus* ATCC BAA-44 showed no susceptibility to any of the antibiotics tested, which confirms its methicillin resistance profile. For the Gram-negative strains, *Escherichia coli* ATCC 25922 showed a resistance to ampicillin, but sensitivity to the other antibiotics tested. Finally, the clinical isolate of *Pseudomonas aeruginosa* showed sensitivity to amikacin and fosfomycin, while demonstrating resistance to the other antibiotics used. The diameters of the growth inhibition zones for the strains tested are shown in Table 1.

#### 2.2.2. Disk Diffusion Test

The antibacterial activity of different synthetized compounds was evaluated in vitro against four strains of pathogenic bacteria via the disk diffusion technique. The outcomes of the preliminary screening test are presented in Table 2. According to the outcomes, only the conjugate **7b** exhibited antibacterial efficacity against the tested Gram-negative strains. Hybrid compound **7b** proved to have remarkable antibacterial activity against *Escherichia coli* strain, with an inhibition zone of 36.4 ± 1.07 mm. This activity is comparable to that of standard antibiotics tested against the same bacterial strain, including cefotaxime (36 mm), imipenem (36 mm), and fosfomycin (40 mm), as well as superior to that of the antibiotic amikacin (34 mm). Compound **7b** also showed antibacterial activity towards *Pseudomonas aeruginosa* with a mean inhibition zone of 11.25 ± 1.02 mm. The pronounced antibacterial properties observed for conjugate **7b** towards the Gram-negative bacterial strains may be attributable to the synergistic effect of the hybridization between two pharmacophores, triazole and isoxazole, particularly when influenced by the presence of chlorine in the ortho position.

#### 2.2.3. Minimum Inhibitory Concentration (MIC) and Minimum Bactericidal Concentration (MBC) Determinations

The MIC and MBC of the hybrid compound **7b** were determined against the pathogenic strains *Escherichia coli* ATCC 25922 and *Pseudomonas aeruginosa*. The obtained outcomes are given in Table 3. The findings reveal that **7b** displayed a selective effect against the tested Gram-negative bacteria, inhibiting their growth at value of MIC of 15 mg/mL for the *Escherichia coli* ATCC 25922, and 30 mg/mL for the *Pseudomonas aeruginosa*. The findings of MBC also substantiate that the hybrid molecule **7b** exhibits a bacteriostatic effect on the strain *Pseudomonas aeruginosa*, while a bactericidal effect on the strain *Escherichia coli* ATCC 25922 is observed at a concentration of 30 mg/mL.

### 2.3. Drug Similarity and ADME-Tox Predictions

In silico investigation applied to the synthesized molecule labeled **7b** reveals that all physicochemical properties satisfy Lipinski’s five rules, such that the molecular weight does not exceed the critical weight of 500 g/mol, the molar refractive index is in the range: 40 ≤ MR ≤ 130, the lipophilicity is given by a partition coefficient of less than 5, and the numbers of hydrogen bond acceptors and donors are less than 10 and 5, respectively, as shown in Table 4. The compound under study is therefore a small molecule that closely resembles drug candidates [50,51,52], Furthermore, ADMET pharmacokinetic characteristics show a desirable profile of the examined compound, substantiated by an excellent human intestinal absorption (HIA of 96.39%), with significant permeability to the central nervous system (CNS) and the blood–brain barrier (BBB) [53]. Moreover, its metabolism on human cytochromes confirm that it is predicted as a potent inhibitor of 2C9, 2C19, and 3A4 cytochromes. Analysis of AMES toxicity shows that the active compound is also predicted as a non-toxic agent which cannot cause any carcinogenic effects on the human body, and does not cause any skin allergies. In contrast, it is predicted to have a positive effect of hepatotoxicity [54,55]. The compound under investigation is expected to have an ADMET profile quite similar to that of compound 7a, as reported in Table 5.

**Figure 2:**
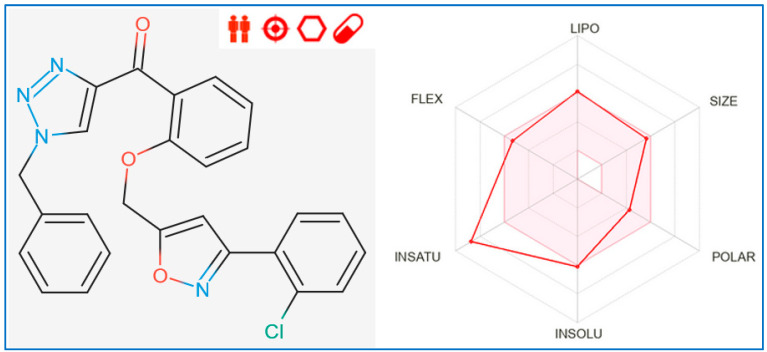
Bioavailability radar of synthesized molecule.

Additionally, the chemical compound under investigation is also predicted to have a good level of oral bioavailability due to the location of the bioavailability radar in the pink area, which is the ideal zone of oral bioavailability in the human body on the basis of six physicochemical characteristics, including flexibility, polarity, lipophilicity, solubility, size, and saturation [56], as shown in Figure 2.

### 2.4. Docking Molecular Study

**Figure 3:**
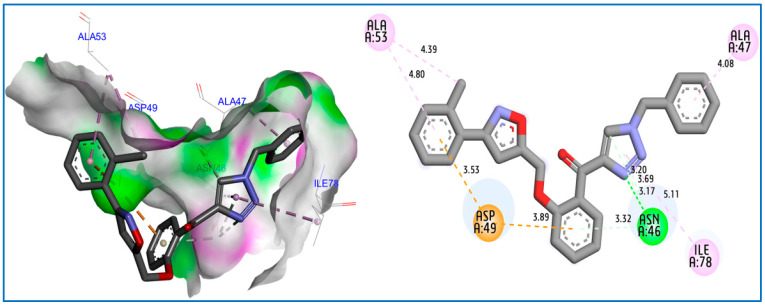

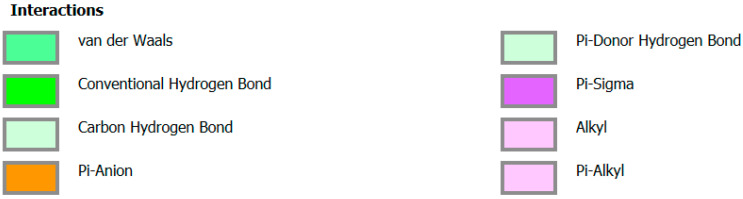
Three- and two-dimensional docking interactions between the molecule **7b** and the targeted protein from *Escherichia coli* pathogenic strain.

To explore the inhibition mechanism with binding energies of the synthesized compound towards the antibacterial proteins from *Escherichia coli* and *Pseudomonas aeruginosa* pathgenic strains, molecular docking simulations were properly carried out, whose the active molecule was initially docked to the crystal structure of an *Escherichia coli* protein (resolution of 2.30 Å) encoded as 1KZN.pdb [57], with a binding energy of −6.22 kcal/mol, forming a variety of intermolecular interactions, including one Hydrogen bond detected with the ASN 46 amino acid residue; one Pi-anion chemical bond with the Asp 49 amino acid residue; and more than three Pi-Alkyl bonds produced towards the Ala 47, Ala 53, and Ile 78 amino acid residues in the A chain, as presented in Figure 3.

**Figure 4:**
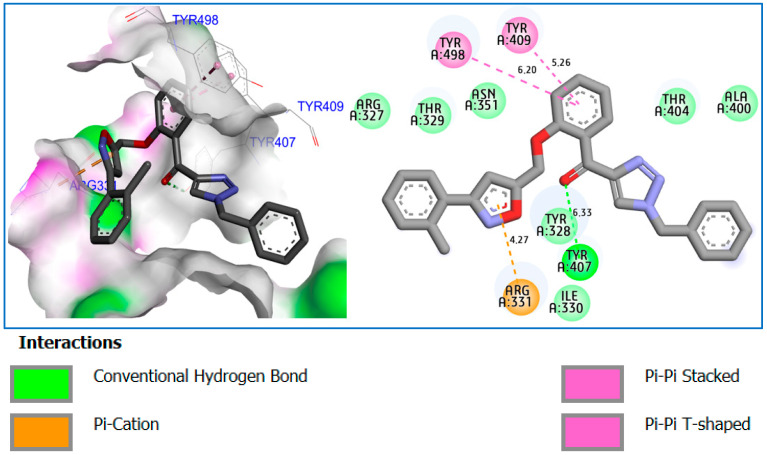
Three- and two-dimensional docking interactions between the synthesized molecule and the targeted protein from the *Pseudomonas aeruginosa* pathogenic strain.

The studied compound was docked for the second time towards the crystal structure of a penicillin-binding protein from *Pseudomonas aeruginosa* (resolution of 2.01 Å) encoded as 4KQR.pdb [58], with a binding energy of −6.05 kcal/mol, producing a family of chemical bonds, including one Hydrogen bond detected towards the Tyr 407 amino acid residue and one Pi-cation bond fixed with the Arg 331 amino acid residue, in addition to two Pi-Pi T-shaped bonds which were created with the Tyr 409 and Tyr 498 amino acid residues in the A chain, as shown in Figure 4.

**Figure 5:**
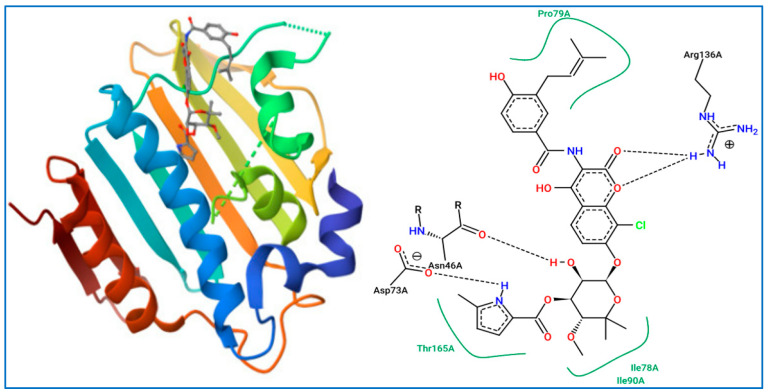
Crystal structure of *Escherichia coli* 24kDa domain protein in complex with clorobiocin (resolution of 2.30 Å), and the active sites of the responsible protein (1KZN.pdb) in A chain.

**Figure 6:**
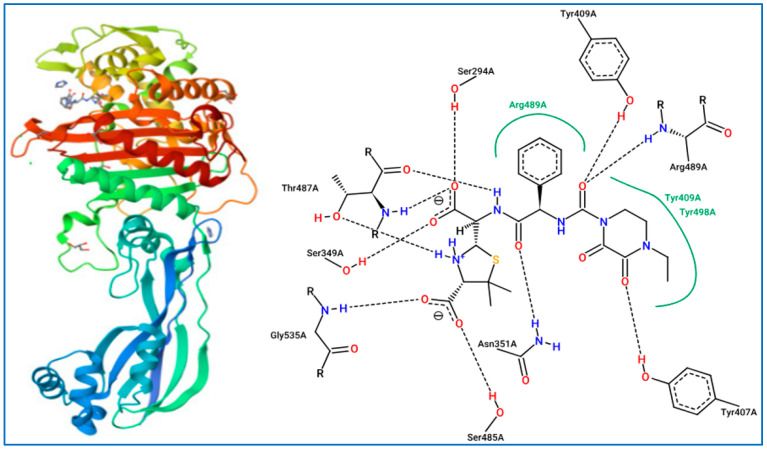
Crystal structure of penicillin-binding protein from *Pseudomonas aeruginosa* in complex with (5S)-penicilloic acid (resolution of 2.01 Å), and the active sites of the responsible protein (4KQR.pdb) in A chain.

The results obtained from two- and three-dimensional visualizations of the intermolecular interactions between the studied ligand and the protein target confirm the validity of two molecular docking processes. The studied compound docked to the active sites of both targeted receptors, with the lowest binding energies exceeding −5.00 kcal/mol where the first complex showed the presence of the amino acid residue ASN 46 as one of the active sites of the protein 1KZN.pdb against the *Escherichia coli* pathogenic strain, as well as the presence of the amino acid residues Tyr 407 and Tyr 409 as two active sites of the 4KQR.pdb protein against the *Pseudomonas aeruginosa* pathogenic strain, as shown in Figure 5 and Figure 6, respectively.

### 2.5. Dynamics Molecular Simulations

The macromolecular complexes of ligand **7b** with the DNA gyrase enzyme of *Escherichia coli* and penicillin-binding protein-3 (PEP3) of *Pseudomonas aeruginosa* were selected for executing MD simulations to evaluate their thermodynamic stability within a specified timeframe of 100 ns. The drug–receptor complex has to be sufficiently stable over a nano-scaler time range to execute the therapeutic response. As a result, each macromolecular complex underwent a 100-ns MD simulation using Schrodinger’s Desmond software version 2022.4. The target DNA gyrase enzyme’s monomeric chain has 186 amino acids consisting of 1446 heavy atoms out of an overall 2879 atoms. Structural alterations and RMSD analysis of the macromolecular backbone was executed during the 100-ns simulation to assess its thermodynamic stability. The complexed ligand **7b** haseight flexible bonds comprising 34 heavy atoms out of 56 atoms in total. The bacterial DNA gyrase–ligand **7b** conjugate shows that the bound ligand demonstrates stabilized conformation throughout the simulation. The RMSD value of the receptor’s backbone was found to fluctuate between 1.2 and 2.1 Å, whereas the bound ligand **7b** exhibited some minor fluctuations to attain stabilized conformation and its RMSD value within receptor’s cavity ranged between 5.0–8.0 Å.

The atoms in a protein or ligand structure might deviate from their initial location, and this can be measured by using their RMSF value. It is a significant parameter for determining the flexibility and dynamic behavior of the macromolecular complex. Protein RMSF is important because it may be used to predict protein dynamics and evaluate stability by providing information about the relative flexibility of various regions. MD-based evaluation of the bacterial DNA gyrase complexed with ligand **7b** has concluded that the RMSF for Cα backbone was found to be within 0.5–2.0 Å with few exceptions, while for ligand **7b** it was found to range from 1.5 to 4.5 Å.

The development of hydrophobic contacts, ionic interactions, and hydrogen bonds during an MD simulation are responsible for the thermodynamic permanence of a receptor–ligand complex and it is evaluated by the continuous monitoring of their strengths throughout the simulation for all the three macromolecular complexes. Throughout the simulation, ligand **7b** was found to be interacting with the bacterial DNA gyrase enzyme via formation of hydrophobic bonds with the amino acids Val43, Arg76, Ile78, Pro79, Ile82, Ala86, Ala87, Val89, Ile90, Met91, Val93, His95, Ala96, His116, Val120, and Leu132, whereas Asn46, His116, and Val120 via hydrogen bonds, while the amino acids Asn46, Asp49, Glu50, Arg76, Gly77, Ile90, His95, Val120, and Ser121 were found to be interacting via water bridges, and amino acid Asp49 was interacting via an ionic bond.

**Figure 7:**
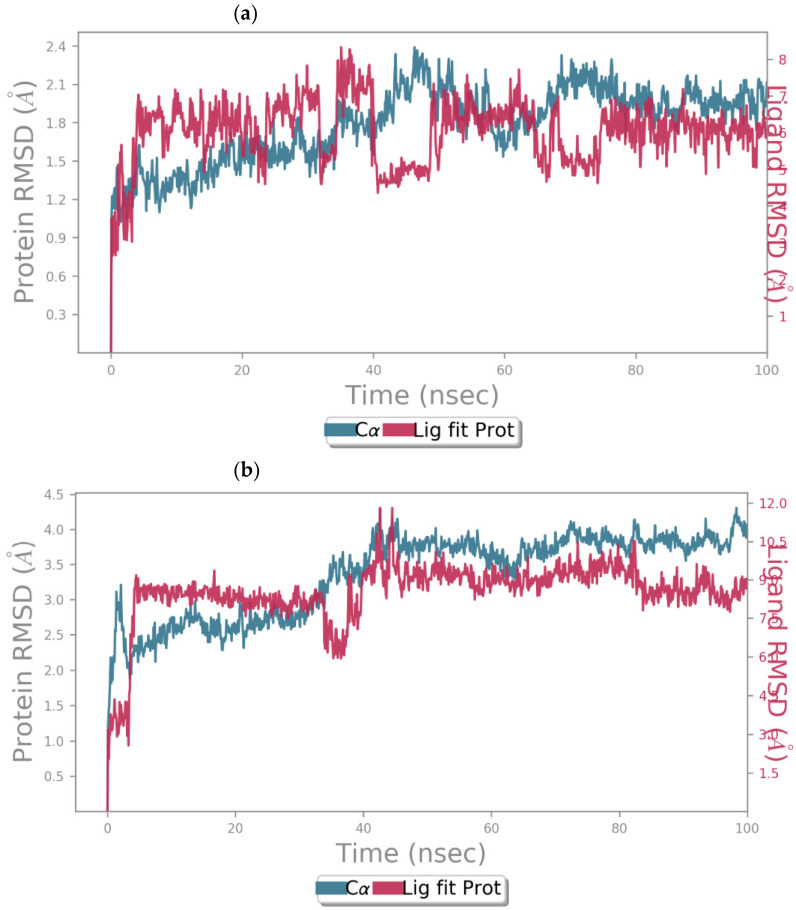
The RMSD for the macromolecular complex of ligand **7b** complexed with DNA gyrase of *Escherichia* coli bacteria (**a**) and PEP3 receptor of *Pseudomonas* aeruginosa (**b**) detected while executing MD simulation.

The target PBP3 receptor’s monomeric chain has 271 amino acids consisting of 2017 heavy atoms out of an overall 4055 atoms. Structural alterations and RMSD analysis of the macromolecular backbone was executed during the 100-ns simulation to assess its thermodynamic stability. The complexed ligand **7b** has eight flexible bonds comprising 34 heavy atoms out of 56 atoms in total. The bacterial PBP3 receptor–ligand **7b** conjugate shows that the bound ligand displays a couple of conformational changes followed by attaining a stabilized conformation. The RMSD value of the receptor’s backbone was found to fluctuate between 2.0 and 4.0 Å, whereas the bound ligand **7b** exhibited some major initial fluctuations to attain stabilized conformation and its RMSD value in receptor cavity, ranging between 6.5 and10.5 Å. Figure 7 demonstrates the revealed RMSD of the ligand **7b** complexed with the DNA gyrase of *Escherichia coli* bacteria (a) and PEP3 receptor of *Pseudomonas aeruginosa* (b), respectively.

**Figure 8:**
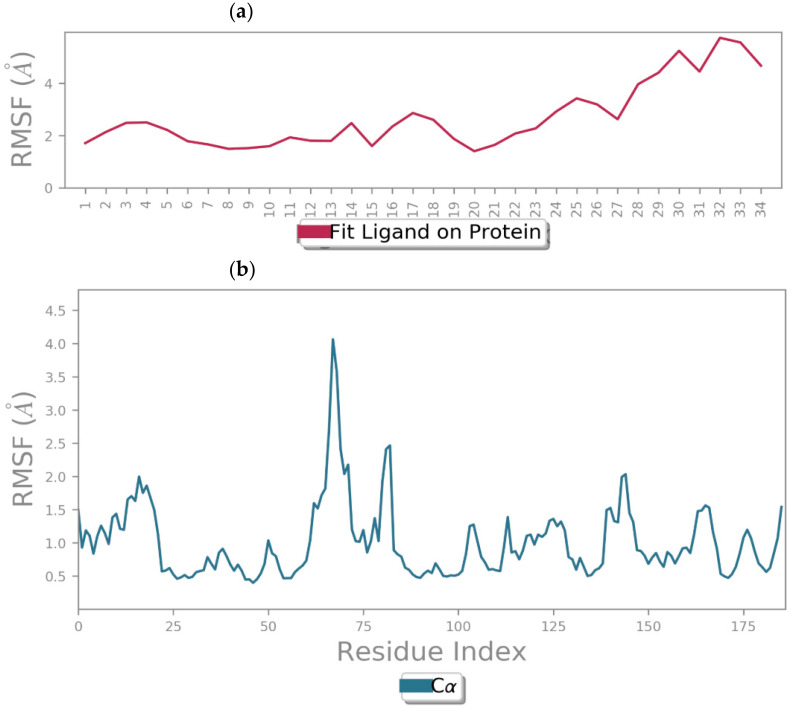
RMSF for DNA gyrase enzyme of *Escherichia coli* (**a**) complexed with ligand **7b** (**b**) detected while executing MD simulation.

**Figure 9:**
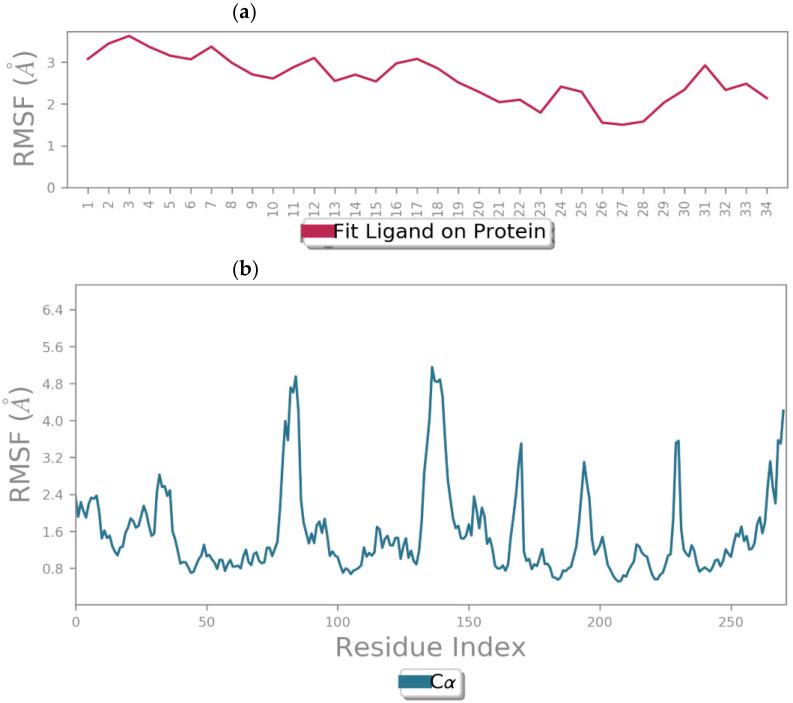
RMSF for PEP3 receptor of *Pseudomonas aeruginosa* (**a**) complexed with ligand **7b** (**b**) detected while executing MD simulation.

MD-based evaluation of bacterial PBP3 receptor complexed with ligand **7b** has concluded that the RMSF for Cα backbone was found to be within 0.8–3.2 Å with few exceptions, while for ligand **7b** it was found to be ranging from 1.5 to 3.5 Å. Figure 8 and Figure 9 shows the RMSF of the ligand **7b** complexed with the DNA gyrase enzyme of *Escherichia coli* (a) and PEP3 receptor of *Pseudomonas aeruginosa* (b), respectively.

**Figure 10:**
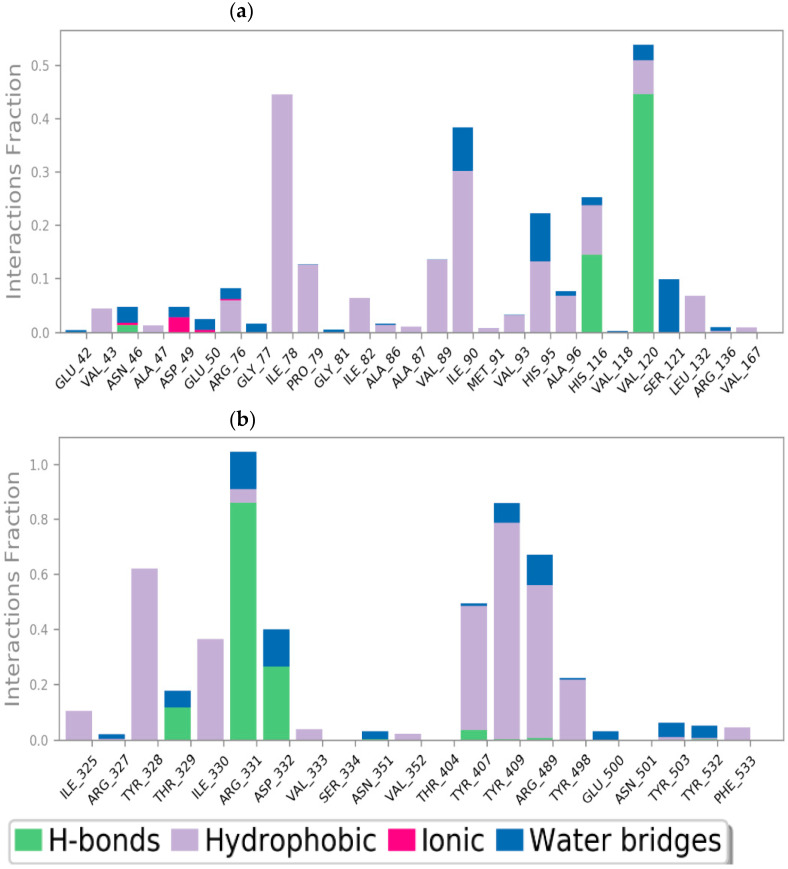
**Protein-ligand contacts:** The ligand **7b** is found to be interacting with the target DNA gyrase enzyme of *Escherichia coli* (**a**), and PEP3 receptor of *Pseudomonas aeruginosa* (**b**) via hydrophobic interactions represented in purple-colored bars, interactions via formation of water bridges represented in blue-colored bars, and hydrogen bonds represented in green-colored bars.

Throughout the simulation, ligand **7b** was found to be interacting with the bacterial PBP3 receptor via formation of hydrophobic bonds with the amino acids Ile325, Tyr328, Ile330, Val333, Tyr407, Tyr409, Arg489, Tyr498, and Phe533, whereas Thr329, Arg331, and Asp332 via hydrogen bonds and amino acid Arg327, Thr329, Arg331, Asp332, Asn351, Arg489, Glu500, Tyr503, andTyr532 were found to be interacting via water bridges. Figure 10 illustrates the interacting residues of the DNA gyrase enzyme of *Escherichia coli* (a) and PEP3 receptor of *Pseudomonas aeruginosa* (b) with the complexed ligand **7b**.

## 3. Materials and Methods

### 3.1. General Information

Full details of the chemicals, solvents and reagents, and equipment utilized in the synthesis of the compounds, alongside the synthesis procedures and characterization data, are provided in the Supplementary Material file.

### 3.2. Evaluation of Antibacterial Activity against Pathogenic Strains

#### 3.2.1. Solution Preparation

Before conducting the experiments, the molecules under investigation were diluted in a dimethyl sulfoxide (DMSO) solution as part of the solution preparation process; the specific goal of this was to generate a stock solution with a concentration of 30 mg/mL. This stock solution was utilized for the disk diffusion tests as well as in MIC and MBC.

#### 3.2.2. Tested Bacterial Strains

Diverse bacterial strains were subjected to testing in this study, comprising three distinct pathogenic bacteria: one Gram-positive cocci and two Gram-negative bacilli. The Gram-positive cocci include two reference strains, namely *Staphylococcus aureus* ATCC 25,923 and *Staphylococcus aureus* ATCC BAA-44. The Gram-negative bacilli consisted of a reference strain of *Escherichia coli* ATCC 25922 and a strain of *Pseudomonas aeruginosa* isolated from the hospital environment (Table 6).

#### 3.2.3. Bacterial Pre-Culture Preparation

Bacterial cultures were derived from pre-existing stock cultures, which had been prepared with 20% (*v*/*v*) glycerol into Brain Heart Infusion (BHI) and storing them at −20 °C. Prior to the tests, a streak plate was prepared on Tryptic Soy Agar (TSA) using these stock cultures and incubated for 24 h at 37 °C. Subsequently, for the experiments, one to two colonies from the aforementioned plate were inoculated into a 0.9% NaCl solution using a sterile loop for McFarland standard adjustment.

#### 3.2.4. Antibiotic Susceptibility Test

Before evaluating the tested molecules, the susceptibility of the bacterial strains to standards drugs was examined using the Kirby–Bauer disk diffusion process [59]. This method classified the strains into sensitive and resistant categories. The determination of antibiotic resistance and selection of appropriate antibiotics for each strain were conducted according to the guidelines of the Antibiogram Committee of the French Society of Microbiology, June 2023 V.1.0 edition (Available online: https://www.sfm-microbiologie.org/wp-content/uploads/2023/06/CASFM2023_V1.0.pdf, accessed on 22 May 2024). Our study utilized a variety of antibiotics, including ampicillin, norfloxacin, cefoxitin, cefotaxime, amikacin, fosfomycin, and imipenem. These antibiotic disks were placed onto Mueller–Hinton agar previously inoculated with a bacterial suspension, followed by an incubation period at 37 °C for 24 h. The concentration per disk of the antibiotics used is detailed in Table 7.

#### 3.2.5. Qualitative and Quantitative Evaluation

(a) Disk diffusion test

The antibacterial action of the examined molecules was determined through the disk diffusion technique [59]. This technique involves utilizing sterile filter paper disks with a diameter of 6 mm, impregnated with the highest concentration employed for each molecule under investigation, specifically 30 mg/mL, at a rate of 10 μL per disk. These disks were positioned on the surface of Mueller–Hinton agar medium, which had been previously inoculated with a bacterial suspension. After being left to rest for 15 min at RT, it was then incubated at 37 °C for 24 h.

(b) MIC determination

To assess the activity of the active molecule, we conducted an experiment to determine the MIC, which is described as the weakest concentration of antibacterial agent needed to prevent the growth of a bacterial species [60]. In our experiment, we determined the MIC using a modified microdilution technique on a microtiter plate described previously by Mammate et al. [61]. A 96-well plate was used in which each well was inoculated with 20 µL of strain inoculated in 0.9% NaCl solution and adjusted to 0.5 McFarland, plus 20 µL of the tested compounds in solutions after performing a 2-fold serial dilution giving us solutions ranging from 30 mg/mL to 0.029 mg/mL for each of the six compounds, plus 120 µL of sterile Mueller–Hinton Broth (MHB). The experiment was conducted 2 to 3 times for each tested molecule. On completion of 24 h incubation at 37 °C, a 20 µL of 0.1% 2,3,5-triphenyltetrazolium chloride (TTC) was introduced, and after a further 4 h incubation at 37 °C, the growth was revealed by the appearance of a reddish color in the wells.

(c) MBC determination

After determining the MIC of the compounds, we conducted a consecutive experiment to evaluate the MBC, which is delineated as the weakest concentration of antibacterial agent necessary to completely kill 99.9% of the final inoculum after incubating for 24 h at 37 °C [62]. According to MIC results, MBC values may be measured after microdilution through the extraction of 100 µL from wells with concentrations at or above MIC, then spreading this volume on Tryptone Soya Agar and observing bacterial growth after incubation for 24 h at 37 °C.

### 3.3. In Silico Studies

In light of the experimental results obtained, which were applied to the synthesized molecule **7b**, revealing its antibacterial potential on pathogenic strains of *Escherichia coli* and *Pseudomonas aeruginosa*, in silico investigations were also carried out to assess its likeness to drug candidates. Initially, physicochemical features were examined based on Lipinski’s five rules. Thereafter, the pharmacokinetic properties of absorption, distribution, metabolism, excretion, and toxicity (ADMET) were equally predicted in the human body. Finally, the inhibition mechanism of this active molecule towards the targeted proteins was studied using the molecular docking technique, in which the produced intermolecular interactions were screened using molecular dynamics simulations.

The physicochemical and ADMET pharmacokinetic characteristics of the studied compound were achieved thanks to the use of pKCSM and SWISS servers. Molecular docking technology was performed using Autodock 4.2 software (Available online: https://mybiosoftware.com/autodock-4-2-3-autodocktools-1-5-6-suite-automated-docking-tools.html, accessed on 22 May 2024), in which the responsible proteins of *Escherichia coli* and *Pseudomonas aeruginosa* strains were extracted from a protein data bank (PDB) basis with 1KZN and 4KQR codes, respectively, then converted from PDB to PDBQT format, and prepared by adding Gasteiger charges and removing water molecules [63,64]. Afterwards, the synthesized compound was docked to the active sites of each targeted protein, and then Discovery Studio software 2021 (Available online: https://www.3ds.com/products-services/biovia/products/molecular-modeling-simulation/biovia-discovery-studio, accessed on 22 May 2024) was used to visualize the intermolecular interactions produced for each complex. At the last stage, molecular dynamics (MD) technology was carried out to examine the thermodynamic stability of the tested compound after being complexed to both responsible proteins throughout 100 nanoseconds of MD simulation time, with the assistance of the Desmond module of Schrodinger’s Maestro software (Available online: (https://www.schrodinger.com/platform/products/maestro/, accessed on 22 May 2024), respecting the standard protocol for preparation, working with OPLS-type force fields, adding water molecules (H_2_O) and counter ions (Na^+^, Cl^−^) at a molar concentration of 0.15 M, and reproducing the same physiological conditions (pressure of 1 atm, and temperature of 300 K) [65].

## 4. Conclusions

To sum up, two new isoxazole–triazole hybrid heterocycles were successfully synthesized employing 3-formylchromone as starting material with good yields. Their structures and those of the intermediate compounds have been thoroughly characterized. The synthesized compounds were screened for their antibacterial activity against three ATCC reference strains as well as one strain isolated from the hospital environment. The findings highlight that conjugate **7b** exhibits a powerful antibacterial effect against *Escherichia coli* ATCC 25922 and *Pseudomonas aeruginosa* strains, compared to standard antibiotics. Moreover, the ADMET pharmacokinetic characteristics revealed a favorable profile for the examined compound, as well as a good level of oral bioavailability. Molecular docking studies supported the antibacterial properties of conjugate **7b** vs. bacterial strains of *Escherichia coli* and *Pseudomonas aeruginosa*, which exhibited elevated binding energies, suggesting strong interactions within bacterial enzyme targets. Additionally, 100-ns MD simulation validated the thermodynamic stability of the complex formed between hybrid compound **7b** and target receptors. Taken together, our comprehensive studies using a combination of synthetic, in vitro, and in silico techniques highlight the promise of triazole–isoxazole conjugates as future antibiotic candidates, particularly hybrid compound **7b**, due to their attractive activity against Gram-negative bacteria.

## Figures and Tables

**Table 1:** Antibiotic susceptibility test results of the studied bacterial strains.

	Strains			
Antibiotic	*Staphylococcus aureus* ATCC 25923	*Staphylococcus aureus* ATCC BAA-44	*Escherichia coli* ATCC 25922	*Pseudomonas aeruginosa*
Ampicillin (AMP)	R (10) *	R (10)	R (0)	R (0)
Norfloxacin (NOR)	S (26)	R (10)	-	-
Cefoxitin (FOX)	S (30)	R (9)	-	R (0)
Cefotaxime (CTX)	-	-	S (36)	R (16)
Imipenem (IMP)	-	-	S (36)	I (30)
Amikacin (AK)	S (21)	R (13)	S (34)	S (24)
Fosfomycin (FOS)	-	-	S (40)	S (20)

R: Resistant; S: Sensitive; I: Intermediate; * Zone of inhibition expected in millimeters (mm).

**Table 2:** Inhibition diameters of the tested molecules. (Each value represents the average of three double disk trials ± SD).

Molecules	*Staphylococcus aureus* ATCC 25923	*Staphylococcus aureus* ATCC BAA-44	*Escherichia coli* ATCC 25922	*Pseudomonas aeruginosa*
**3′**	-	-	-	-
**3**	-	-	-	-
**6**	-	-	-	-
**7a**	-	-	-	-
**7b**	-	-	36.4 ± 1.07 *	11.25 ± 1.02

* Zone of inhibition expected in millimeters (mm).

**Table 3:** MIC and MBC of the tested molecule expressed in mg/mL.

Compound	Test	*Staphylococcus aureus* ATCC 25923	*Staphylococcus aureus* ATCC BAA-44	*Escherichia coli* ATCC 25922	*Pseudomonas aeruginosa*
**7b**	MIC	-	-	15	30
	MBC	-	-	30	-

**Table 4:** Prediction of physicochemical properties of the molecule **7b**.

Studied Compound	Physicochemical Properties					Lipinski Rules
	Molecular Weight (g/mol)	Molar Refractive Index	Log P (Octanol/Water)	Hydrogen Bonds Acceptors	Hydrogen Bonds Donors	Categorical (Yes/No)
Rule	≤500	40 ≤ MR ≤ 130	<5	≤10	<5	
**7b**	470.91	127.13	3.67	6	0	YES

**Table 5:** Prediction of the pharmacokinetic properties for the active molecule **7b**.

Studied Compound	A	D		M							E	T		
	Human Intestinal Absorption	Blood–Brain Barrier Permeability	Central Nervous System Permeability	Substrate		Inhibitor					Total Clearance	AMES Test of Toxicity	Hepatotoxicity	Skin Sensitization
				Cytochromes										
				2D-6	3A-4	1A-2	2C-19	2C-9	2D-6	3A-4				
	(% Absorbed)	(Log BB)	(Log PS)	(No/Yes)							Numeric (Log mL/min/kg)	Categorical (No/Yes)		
**7a**	94.946	−1.465	−2.06	No	Yes	No	Yes	Yes	No	Yes	0.039	No	Yes	No
**7b**	96.39	−1.199	−2.097	No	Yes	No	Yes	Yes	No	Yes	0.34	No	Yes	No

A: Absorption; D: Distribution; M: Metabolism; E: Excretion; T: Toxicity.

**Table 6:** Tested bacterial strains.

Pathogen Type	Pathogen Species	Strain
Gram-positive	*Staphylococcus aureus*	ATCC 25923
		ATCC BAA-44
Gram-negative	*Escherichia coli*	ATCC 25922
	*Pseudomonas aeruginosa*	Clinical isolate

**Table 7:** Description of the antibiotics used in susceptibility test.

Antibiotic Class	Antibiotic	Concentration (µg/disk)
Penicillin	Ampicillin	25
Fluoroquinolones	Norfloxacin	10
Cephalosporines	Cefoxitin	30
	Cefotaxime	30
Carbapenem	Imipenem	30
Aminosides	Amikacin	30
Fosfomycin	Fosfomycin	200

## Data Availability

All additional data analyzed during this study can be found in the “Supplementary Materials” section of this article.

## Supplementary Materials

The following supporting information can be downloaded at: https://www.mdpi.com/article/10.3390/molecules29112510/s1, Figure S1: IR spectrum of compound **3**; Figure S2: ^1^H NMR spectrum (300 MHz, CDCl_3_) of compound **3**; Figure S3: ^13^C NMR spectrum (75 MHz, CDCl_3_) of compound **3**; Figure S4: Mass spectrum of compound **3**; Figure S5: IR spectrum of compound **3′**; Figure S6: ^1^H NMR spectrum (300 MHz, CDCl_3_) of compound **3′**; Figure S7: ^13^C NMR spectrum (75 MHz, CDCl_3_) of compound **3′**; Figure S8: Mass spectrum of compound **3′**; Figure S9: IR spectrum of compound **6**; Figure S10: ^1^H NMR spectrum (600 MHz, CDCl_3_) of compound **6**; Figure S11: ^13^C NMR spectrum (150 MHz, CDCl_3_) of compound **6**; Figure S12: Mass spectrum of compound **6**; Figure S13: IR spectrum of compound **7a**; Figure S14: ^1^H NMR spectrum (600 MHz, CDCl_3_) of compound **7a**; Figure S15: ^13^C NMR spectrum (150 MHz, CDCl_3_) of compound **7a**; Figure S16: Mass spectrum of compound **7a**; Figure S17: IR spectrum of compound **7b**; Figure S18: ^1^H NMR spectrum (600 MHz, CDCl_3_) of compound **7b**; Figure S19: ^13^C NMR spectrum (150 MHz, CDCl_3_) of compound 7b; Figure S20: Mass spectrum of compound **7b**.
