# Supplementary material for: New Triazole-Isoxazole Hybrids as Antibacterial Agents: Design, Synthesis, Characterization, In Vitro, and In Silico Studies
Source: Molecules. 2024 May 26;29(11):2510. doi: 10.3390/molecules29112510 (PMC11174038; doi:10.3390/molecules29112510)
Supplement: Supplementary file 1 [file molecules-29-02510-s001.zip › molecules-3016603-supplementary.pdf]

Supplementary Material

New Triazole-Isoxazole Hybrids as Antibacterial Agents: Design, Synthesis, Characterization, In Vitro, and In Silico Studies

Rachid Bouzammit [1,*], Salim Belchkar [2], Mohamed El fadili [3], Youssra Kanzouai [1], Somdutt Mujwar [4], Mohammed M. Alanazi [5], Mohammed Chalkha [1,6], Asmae Nakkabi [1,6], Mohamed Bakhouch [7], Emese Gal [8], Luiza Ioana Gaina [8] and Ghali al houari [1]

[1] Engineering Laboratory of Organometallic and Molecular Materials and Environment, Faculty of Sciences Dhar EL Mahraz, University Sidi Mohamed Ben Abdellah, P.O. Box 1796, Atlas, Fez 30000, Morocco; youssra.kanzouai@usmba.ac.ma (Y.K.); mohammed.chalkha1@usmba.ac.ma (M.C.); asmaenakkabi@yahoo.fr (A.N.); ghalialhouari@gmail.com (G.a.h.)

[2] Laboratory of Epidemiology and Research in Health Sciences, Faculty of Medicine, Pharmacy and Dentistry, University Sidi Mohammed Ben Abdellah, Fez 30070, Morocco; salim.belchkar@usmba.ac.ma

[3] LIMAS Laboratory, Chemistry Department, Faculty of Sciences Dhar EL Mahraz, Sidi Mohamed Ben Abdellah University, P.O. Box 1796, Atlas, Fez 30000, Morocco; mohamed.elfadili@usmba.ac.ma

[4] Chitkara College of Pharmacy, Chitkara University, Rajpura 140401, Punjab, India; somdutt.mujwar@chitkara.edu.in

[5] Department of Pharmaceutical Chemistry, College of Pharmacy, King Saud University, Riyadh 11451, Saudi Arabia; mmalanazi@ksu.edu.sa

[6] Laboratory of Materials Engineering for the Environment and Natural Ressources, Faculty of Sciences and Techniques, University of Moulay Ismail of Meknès, B.P 509, Boutalamine, Errachidia 52000, Morocco

[7] Bioorganic Chemistry Team, Faculty of Science, Chouaïb Doukkali University, P.O. Box 24, El Jadida 24000, Morocco; bakhouch.m@ucd.ac.ma

[8] Research Center on Fundamental and Applied Heterochemistry, Faculty of Chemistry and Chemical Engineering, Babeş-Bolyai University, 11 Arany Janos Str., RO-400028 Cluj-Napoca, Romania; emese.gal@ubbcluj.ro (E.G.); gaina.ioana.luiza@gmail.com (L.G.I.)

* Correspondence: rachid.bouzammit@usmba.ac.ma

1. General Information

All chemicals utilized were of commercial and analytical quality, obviating the need for additional purification steps. Thin-layer chromatography (TLC) served as a means to monitor reaction progression, employing Merck silica gel 60 F254 plates. Visualization of TLC plates was facilitated through UV light exposure using a VILBER LOURMAT VL-215.LC apparatus. Melting point determinations were conducted with precision using a KOFLER bench, achieving an accuracy level of ±2°C. Infrared (IR) spectra were acquired and analyzed in terms of wavenumbers utilizing a BRUKER VERTEX 70 FT-IR spectrometer. Solution-state 1D NMR spectra, both proton (^{1}H) and carbon (^{13}C), were recorded at ambient temperature employing Bruker Avance instruments operating at 300 MHz/75 MHz and 600 MHz/150 MHz, respectively, and utilizing deuterated solvents ($CDCl_3$ or DMSO). Chemical shifts in NMR spectra are expressed in parts per million (ppm). The Attached Proton Test (APT) experiment, incorporated into the ^{13}C NMR spectra, provided insights into the number of carbon signals, distinguishing between CH_3, CH_2, CH, and quaternary carbon (Cq) signals. CH_3 and CH carbon signals may appear as negative or positive depending on the specific spectra, as do CH_2 and quaternary carbon signals. High-resolution mass spectrometry (HRMS) data were acquired using a Thermo Scientific LTQ Orbitrap XL instrument.

2. Synthesis Procedures and Characterization Data

2.1 Synthesis Method for Compounds 3 and 3′

In a round-bottom flask equipped with a reflux condenser, 1 equivalent of 4-oxo-4H-chromene-3-carbaldehyde and 2 equivalents of azidomethylbenzene were introduced into 30 mL of toluene. The reaction mixture was then heated for 10 days, and the progress of the reaction was monitored by thin-layer chromatography (TLC). Once the reaction was complete, the solvent was evaporated under reduced pressure, and the resulting residue was separated by column chromatography using a hexane/ethyl acetate mixture (9/1).

(1-benzyl-1H-1,2,3-triazol-4-yl)(2-hydroxyphenyl)methanone 3

Solid White, m.p. = 104 °C, Yield 11% ; IR ($CHCl_3$, ν in cm^{-1}**)** 3150 (OH), 1600 (C=O, Ketone); ^{1}H NMR ($CDCl_3$, δ in ppm: δ=12.37 (s, 1H, OH), 9.19 (dd, 1H, Ar-H, ^{3}J = 8.70 Hz, ^{4}J = 1.80 Hz), 8.20 (s, 1H**,** $H_{triazole}$), 7.54 (tt, 1H, ^{3}J = 7.80 Hz, ^{3}J= 7.65 Hz, ^{4}J = 1.50 Hz), 6.99–7.58 (m, 7H, Ar-H), 5.63 (s, 2H, -NCH_2), ^{13}C NMR ($CDCl_3$, δ in ppm): 188.89 (<u>C</u>=O), 163.90 (<u>C</u>-OH), 148.37 (<u>C</u>$_{4triazole}$**)**, 136.8, 133.74, 133.53, 129.43, 129.30, 128.76, 128.42, 119.28, 118.08, 54.54 (<u>C</u>H_2). HRMS (*m*/*z*) $[C_{16}H_{13}N_3O_2+H]^+$: calculated Mass: 280,10805, found Mass: 280.10694.

2-(benzylamino)-4-oxo-4H-chromene-3-carbaldehyde 3'

Solid yellow, m.p. = 174 °C, Yield 90%; IR ($CHCl_3$, *v* in cm^{-1}) 1615 (C=O, Ketone); 1615 (CH=O); 3100 (N-H); ^{1}H NMR ($CDCl_3$, δ in ppm): 10.95 (s, 1H, N-H), 10.27 (s, 1H, CH=O), 8.25 (dd, 1H, Ar-H, ^3J = 7.8 Hz, ^4J = 1.2 Hz), 7.63 (tt, 1H, Ar-H, ^3J = 7.8 Hz, ^3J = 0.9 Hz, ^4J = 1.80 Hz), 7.28–8.45 (m,7H, Ar-H), 4.80 (d, 2H, CH_2, ^3J = 6 Hz); ^{13}C NMR ($CDCl_3$, δ in ppm): 189.62 (CH=O), 175.77 (C=O, Ketone), 164.35 (C_2), 153.32, 135.78, 133.69, 129.13, 128.30, 127.49, 126.38, 125.80, 122.88, 116.58, 99.52 (C_3), 44.95 (CH_2). HRMS (*m/z*) $[C_{17}H_{13}NO_3+H]^+$: calculated mass: 280.09682, found mass: 280.09592.

2.2 (1-benzyl-1H-1,2,3-triazol-4-yl)(2-(prop-2-yn-1-yloxy)phenyl)methanone 6

To prepare the propargylic triazole 6, compound 3 (1 equivalent) was dissolved in 10 mL of DMF, then K_2CO_3 (2 equivalents) was added. The mixture was stirred at room temperature until the starting material disappeared, then propargyl bromide (1.2 equivalents) was added dropwise. Once the reaction was complete, the resulting mixture was quenched with 50 mL of ice-cold water. The formed solid was filtered, dried, and recrystallized in ethanol.

Solid brown, m.p. = 134 °C, Yield 56%; IR ($CHCl_3$, *v* in cm^{-1}) 3300 (≡C-H); 1660 (C=O, Ketone); ^{1}H NMR ($CDCl_3$, δ in ppm); δ=8.20 (s, $1H_{triazole}$), 7.58 (dd, 1H, Ar-H, ^3J = 7.65 Hz, ^4J = 1.74 Hz), 7.47 (tt, 1H, Ar-H, ^3J = 6.54 Hz ,^4J = 5.64 Hz), 7.35 (m, 3H, Ar-H), 7.29 (m, 2H, Ar-H), 7.15 (dd, 1H, Ar-H, ^3J = 8.46 Hz , ^4J = 0.96 Hz), 7.08 (tt, 1H, Ar-H, ^3J = 8.4 Hz , ^4J = 3.94 Hz, ^4J = 0.96 Hz), 5.55 (s, 2H, N-CH_2), 4.63 (d, 2H, O-CH_2, ^4J = 2.4 Hz), 2.41 (t, 1H, ≡CH, ^4J = 2.46 Hz), ^{13}C NMR ($CDCl_3$ δ in ppm); δ=187.34 (C=O), 158.38 (C-OH), 148.37 ($C_{4triazole}$), 133.94, 132.75, 130.47, 129.28, 128.33($C_{5triazole}$), 129.06, 127.37, 113.68, 77.14 (-C≡), 75.92 (≡CH), 56.66 (N-CH_2), 54.36 (O-CH_2), HRMS (*m/z*) $[C_{19}H_{15}N_3O_2+H]^+$: calculated Mass: 318.12370, found Mass: 318,12485.

2.3 Hybrid Compounds 7a–b

To a **6** (1eq.) solution in 15 mL of dichloromethane, arylhydroxamoyl bromides (1.2 equivalents) were added with stirring. After 20 minutes, three drops of triethylamine were added to the mixture. The reaction progress was monitored by TLC. The mixture was then extracted with CH_2Cl_2, evaporated and dried. The resulting solid was recrystallized in ethanol.

(1-benzyl-1H-1,2,3-triazol-4-yl)(2-((3-(4-(trifluoromethyl)phenyl)isoxazol-5-yl)methoxy)phenyl)methanone 7a

Solid white, m.p. = 191 °C, Yield 81%; IR ($CHCl_3$, *v* in cm^{-1}) 1655 (C=O, ketone); ^{1}H NMR ($CDCl_3$, δ in ppm); δ=8.07 (s,$1H_{triazole}$), 7.92 (dd, 2H, Ar-H, ^3J = 8.4 Hz, ^4J = 0.6 Hz), 7.74 (d, 2H, Ar-H, ^2J = 8.04 Hz), 7.66 (dd, 1H, Ar-H, ^3J = 7.62 Hz, ^4J = 1.8 Hz), 7.54 (tt, 1H, Ar-H, ^3J = 9.18 Hz, ^4J = 2.46, Hz, ^4J = 1.74 Hz), 7.35-7.38 (m,3H, Ar-H), 7.18 (tt, 2H, Ar-H, ^3J = 8.4 Hz , ^4J = 1.75 Hz, ^4J = 0.9 Hz), 7.10 (dd, 2H, Ar-H, ^3J = 8.28 Hz , ^4J = 0.78 Hz), 6.64 (s, $1H_{isoxazole}$), 5.58 (s, N-$\underline{C}H_2$), 5.26 (s, O-$\underline{C}H_2$); ^{13}C NMR ($CDCl_3$, δ in ppm); δ=187.52 ($\underline{C}$=O), 163.71 (=$\underline{C}$-O), 161.35 ($\underline{C}_{3isoxazole}$), 133.58 ($\underline{C}_{5isoxazole}$), 133.01, 132.17, 131.86, 130.64, 129.34, 129.18, 128.90, 128.32, 127.21, 126.98, 125.98 (q, CF_3), 125.96, 125.91, 125.93, 122.10 ($\underline{C}_{5triazole}$), 101.49 ($\underline{C}H_{isoxazole}$), 62.33 (N-$\underline{C}H_2$), 54.36 (O-$\underline{C}H_2$). HRMS (*m/z*) $[C_{27}H_{19}F_3N_4O_3+H]^+$: calculated Mass: 505.14820, found Mass: 505.14828.

1-benzyl-1H-1,2,3-triazol-4-yl)(2-((3-(2-chlorophenyl)isoxazol-5-yl)methoxy)phenyl)methanone 7b.

Brown liquid, Yield 67%, IR ($CHCl_3$, *v* in cm^{-1}) 1670 (C=O, ketone); ^{1}H NMR ($CDCl_3$, δ in ppm); δ = 8.05 (s,$1H_{triazole}$), 7.72 (dd, Ar-H, ^3J = 8.28 Hz , ^4J = 1.86 Hz), 7.62 (dd, Ar-H, ^3J = 7.56 Hz, ^4J = 1.74 Hz), 7,54 (tt, Ar-H, ^3J = 9.18 Hz , ^4J = 1.74 Hz, ^4J = 0.96 Hz), 7.55 (dd, Ar-H, ^3J = 7.98 Hz , ^4J = 1.44 Hz), 7.43 (tt, Ar-H, ^3J = 9.78 Hz, ^4J = 1.92 Hz, J = 0.83 Hz), 7.42 (dd, Ar-H, ^3J = 7.5 Hz, ^4J = 1.44 Hz), 7.36 (m, Ar-H), 7.30 (d, Ar-H, ^2J = 6.72 Hz), 7.16 (tt, Ar-H, ^3J = 8.4 Hz, ^4J = 1.87 Hz ,^4J = 0.9 Hz), 7.12 (d, Ar-H, ^3J = 8.76 Hz), 6.64 (s, $1H_{isoxazole}$), 5.58 (s, N-CH_2), 5.26 (s, O-CH_2); ^{13}C NMR ($CDCl_3$ δ in ppm); δ=187.91 ($\underline{C}$=O), 161.34 (=$\underline{C}$-O), 156.51 ($\underline{C}_{3isoxazole}$), 148.74 ($\underline{C}_{5isoxazole}$), 113.80, 134.61, 133.96, 133.28, 133.22, 132.08, 131.31, 130.82, 130.76, 130.24, 129.76, 129.61, 129.42, 129.32, 128.70, 127.46, 127.23, 122.37 ($\underline{C}_{5triazole}$), 104.99 ($\underline{C}H_{isoxazole}$), 62.66 (N-$\underline{C}H_2$), 54.72 (O-$\underline{C}H_2$). HRMS (*m/z*) $[C_{26}H_{19}ClN_4O_3+H]^+$: calculated Mass: 471.12184, found Mass: 471,12320.

3. Copies of ^{1}H, ^{13}C NMR, and HRMS Spectra of Compounds

3.1 Spectra of (1-benzyl-1H-1,2,3-triazol-4-yl)(2-hydroxyphenyl) methanone

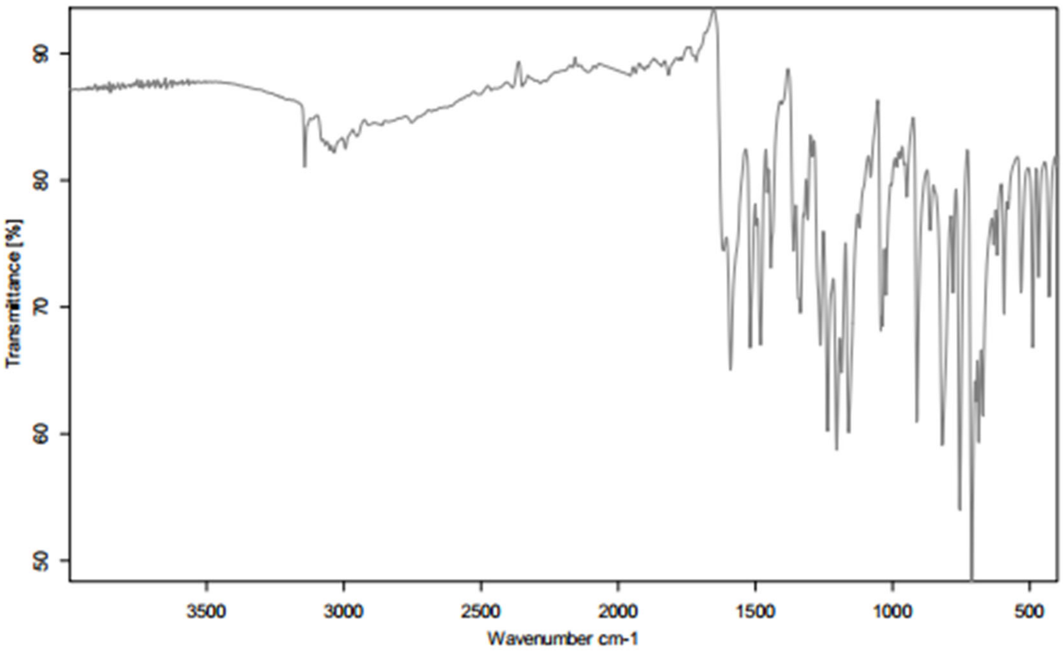

Figure S1. IR spectrum of compound **3.**

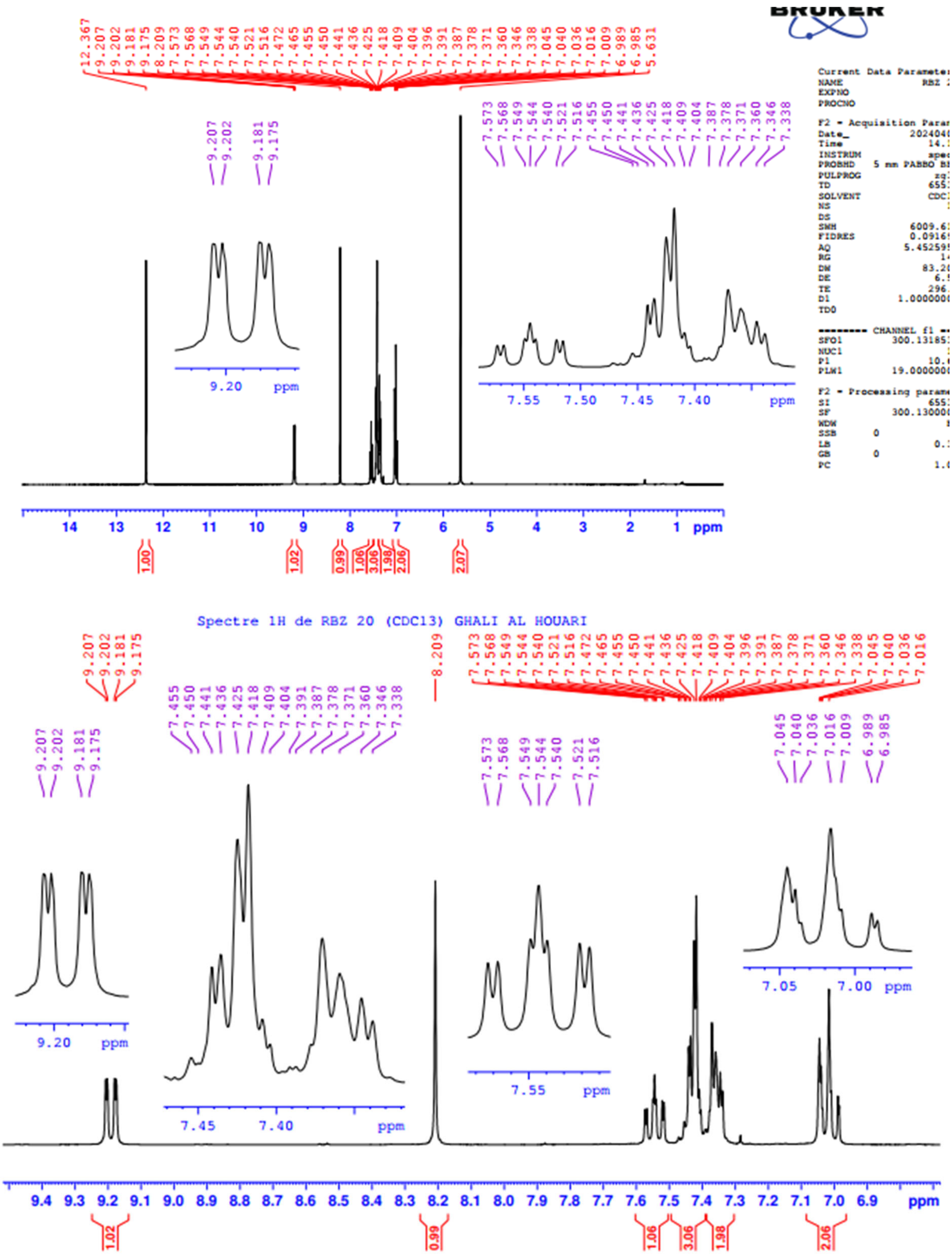

Figure S2. ^{1}H NMR spectrum (300 MHz, $CDCl_3$) of compound **3**.

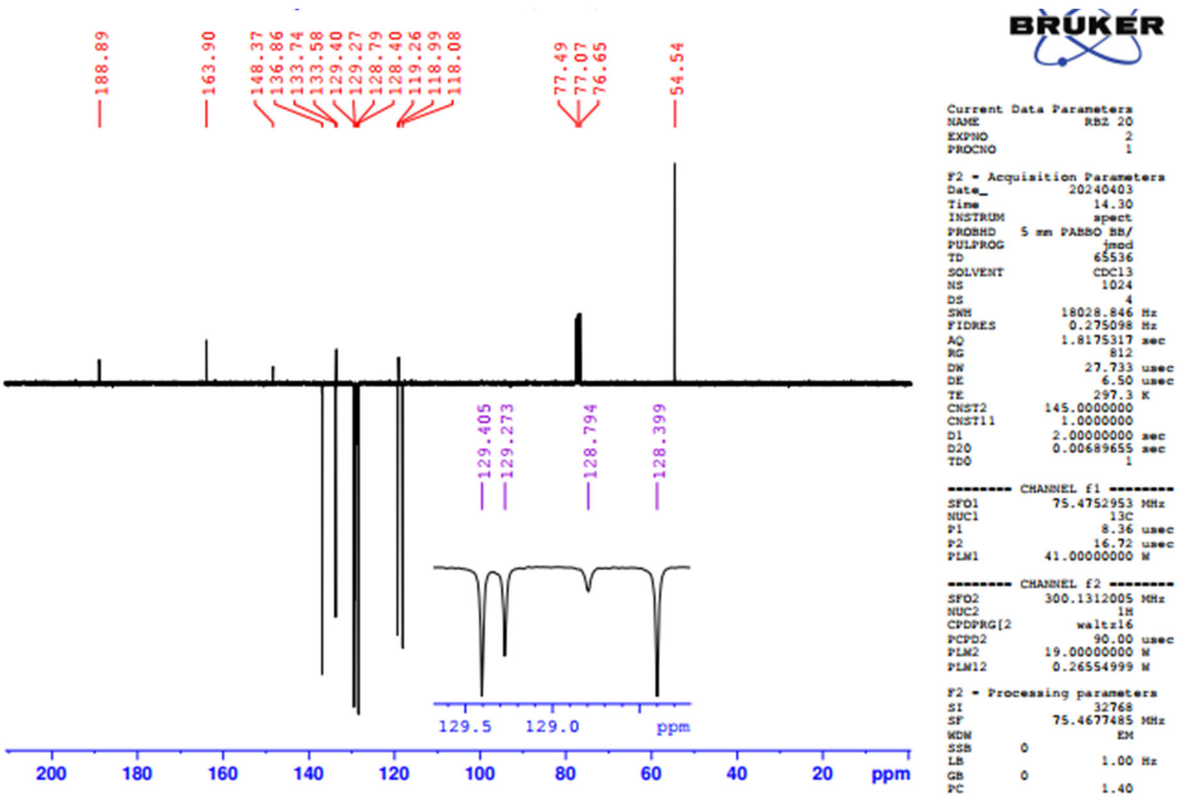

Figure S3. ^{13}C NMR spectrum (75 MHz, $CDCl_3$) of compound **3.**

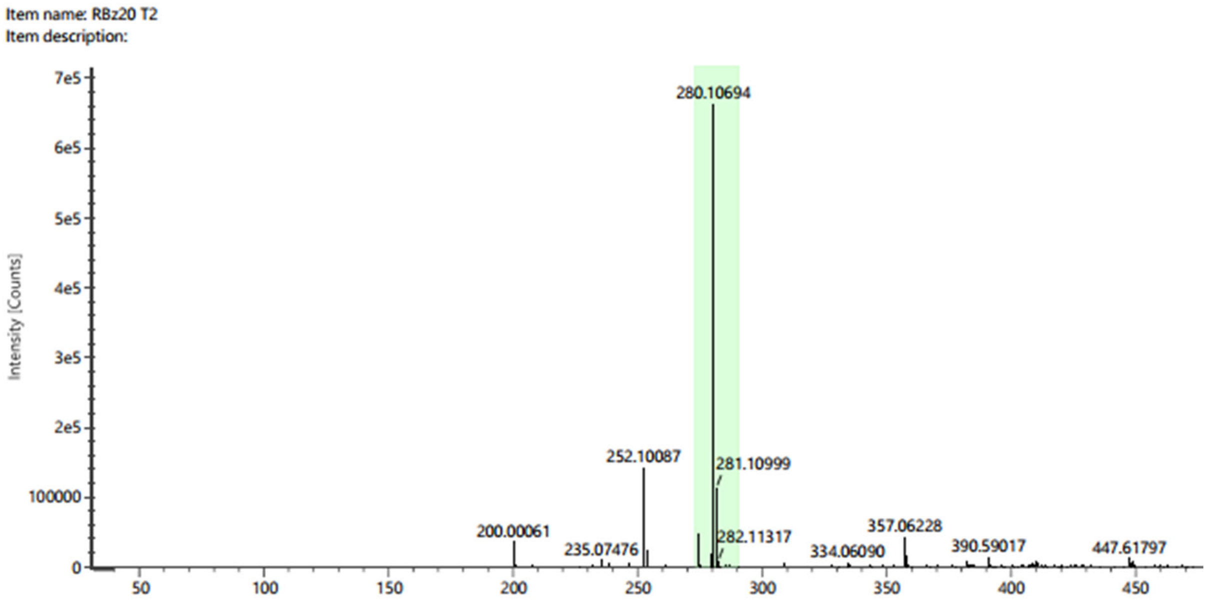

Figure S4. Mass spectrum of compound **3**.

3.2 Spectra of 2-(benzylamino)-4-oxo-4H-chromene-3-carbaldehyde

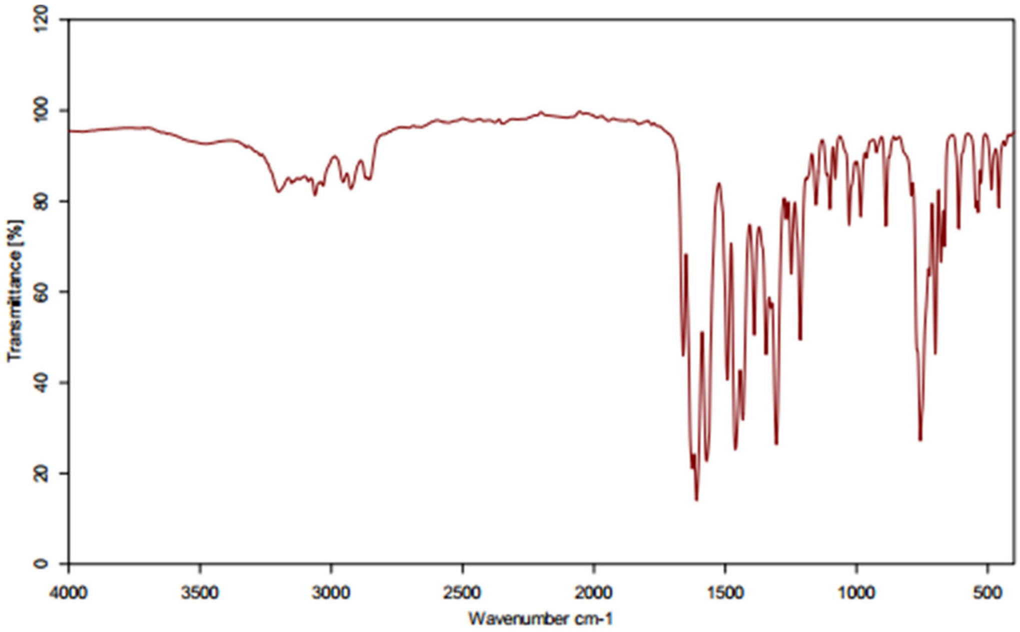

Figure S5. IR spectrum of compound **3'**.

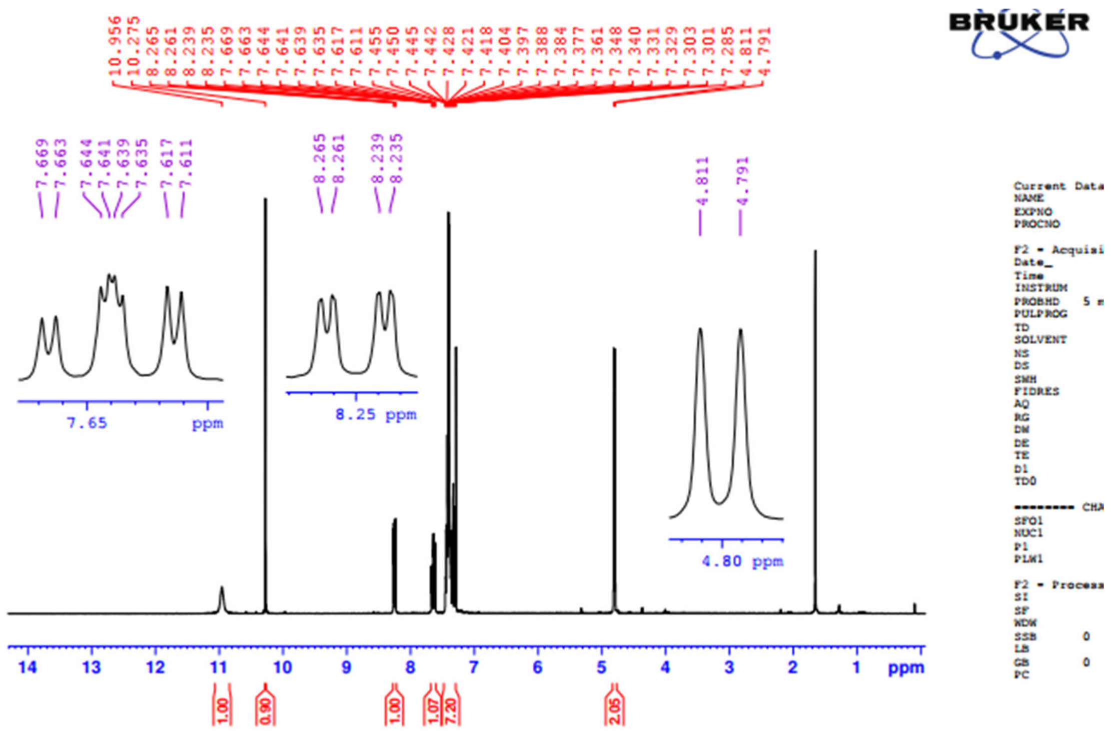

Figure S6. ^{1}H NMR spectrum (300 MHz, $CDCl_3$) of compound **3'**.

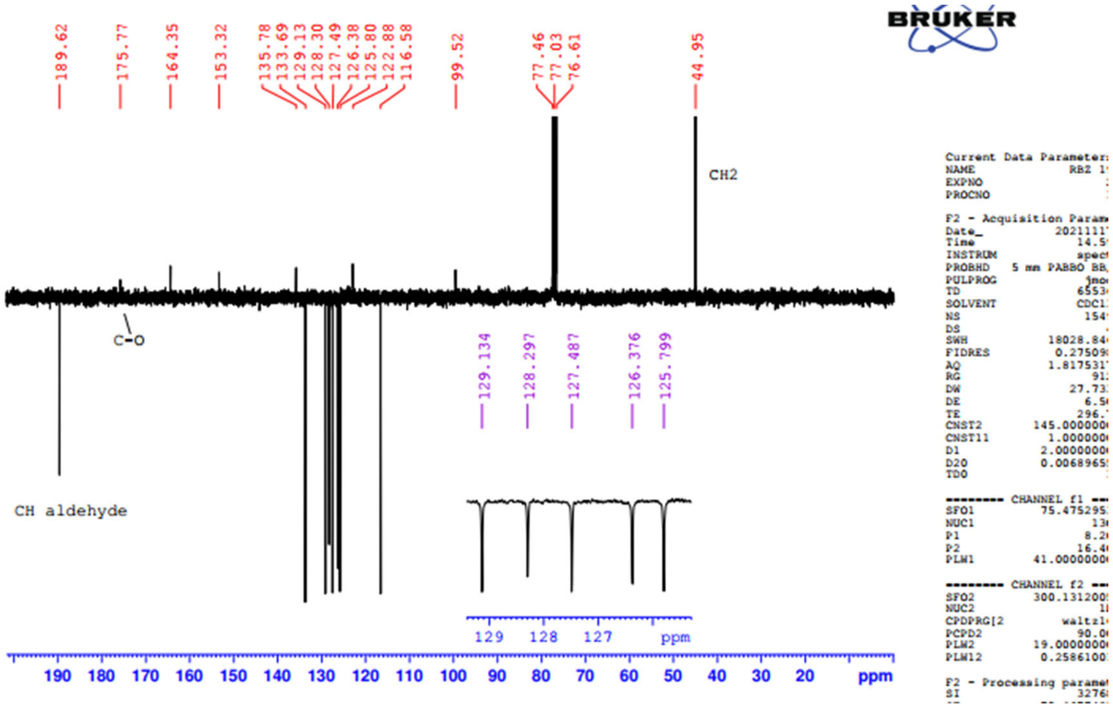

Figure S7. ^{13}C NMR spectrum (75 MHz, $CDCl_3$) of compound **3’**.

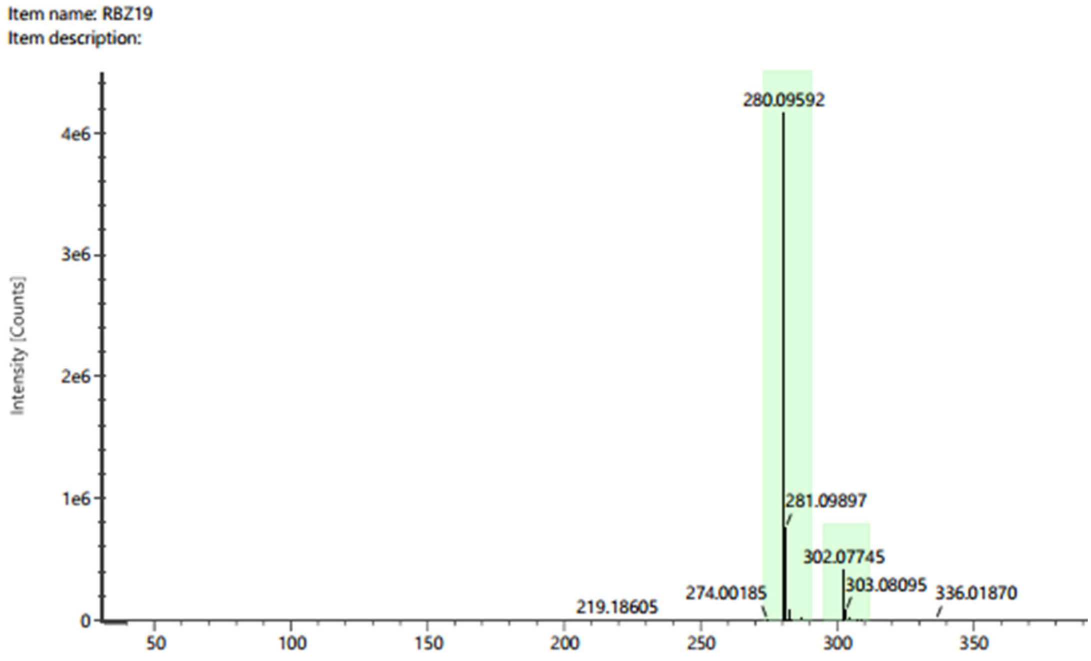

Figure S8. Mass spectrum of compound **3’**.

3.3. Spectra of dipolarophile 6

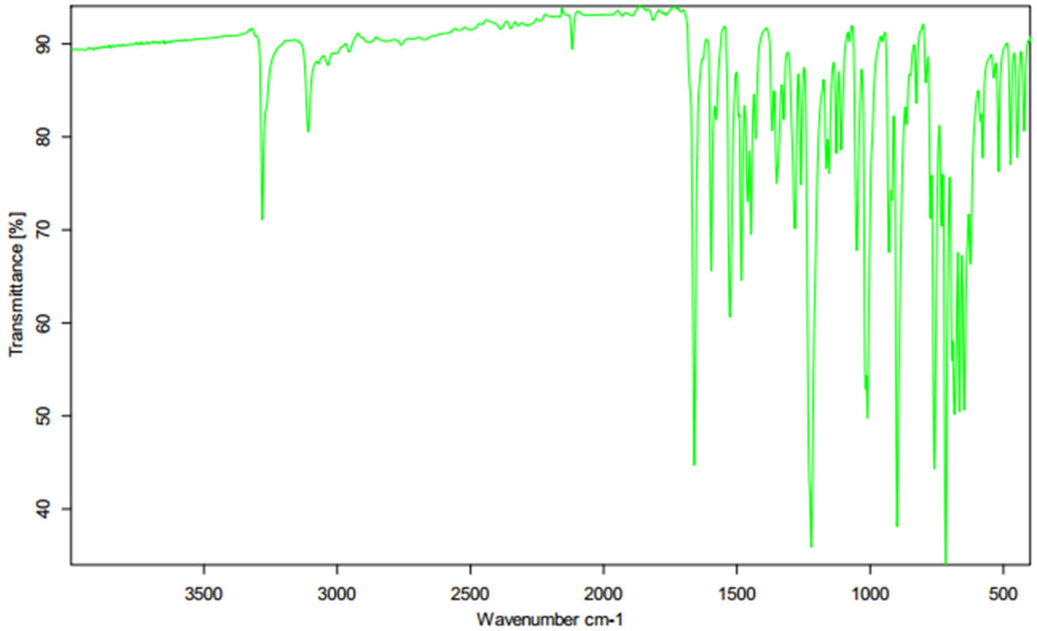

Figure S9. IR spectrum of compound **6**.

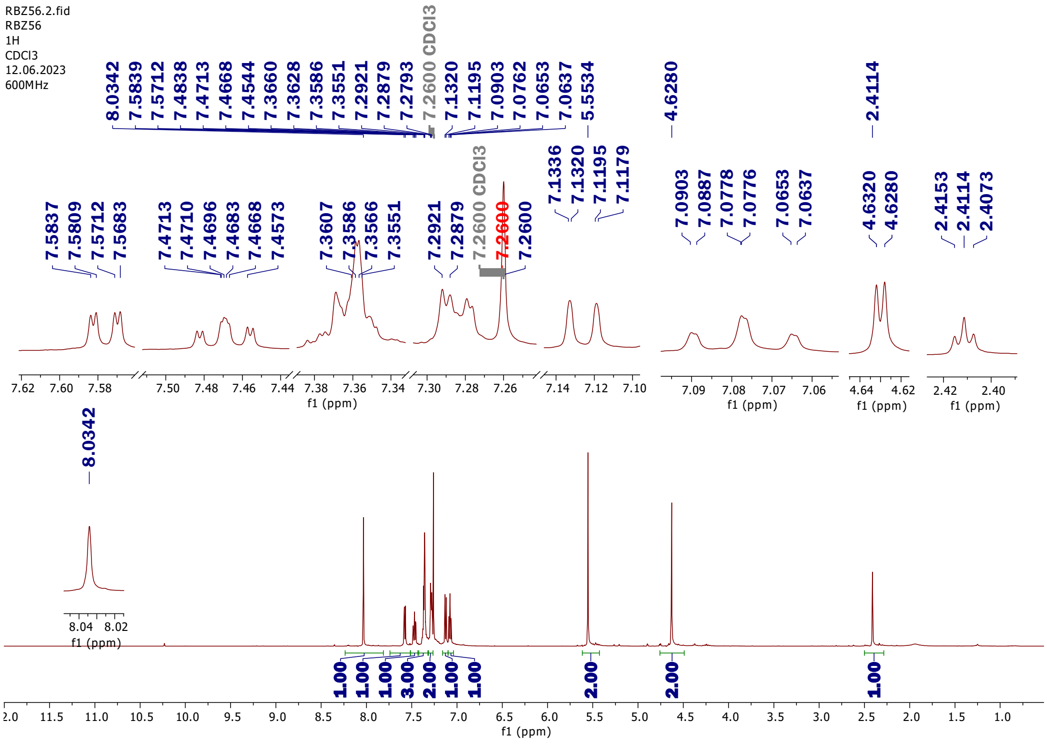

Figure S10. ^{1}H NMR spectrum (600 MHz, $CDCl_3$) of compound **6.**

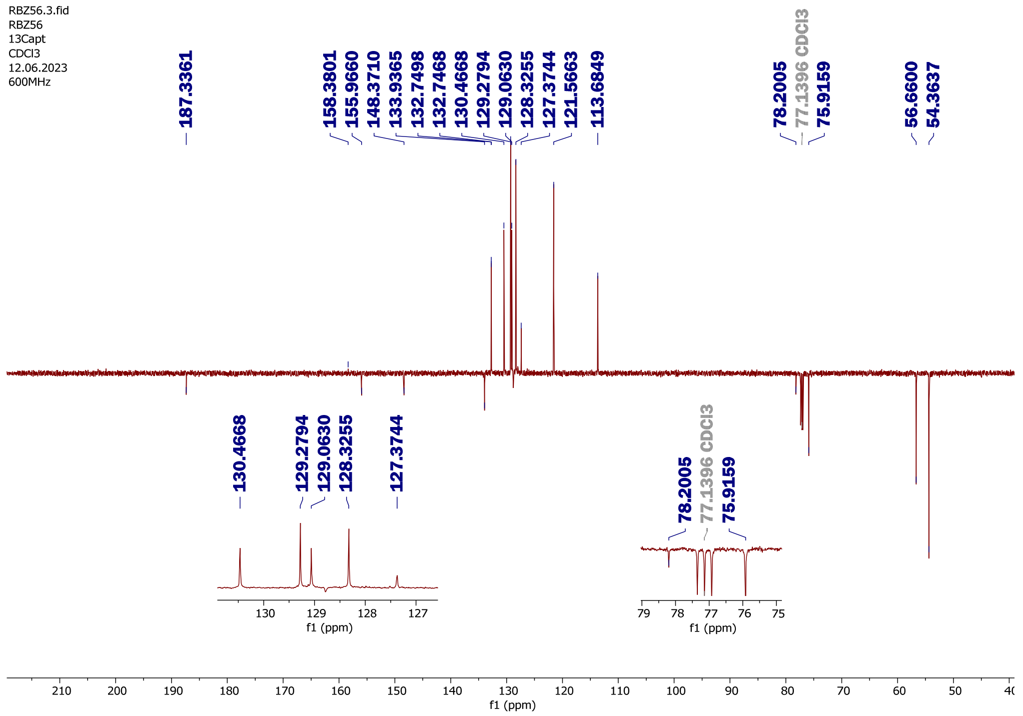

Figure S11. ^{13}C NMR spectrum (150 MHz, $CDCl_3$) of compound **6**.

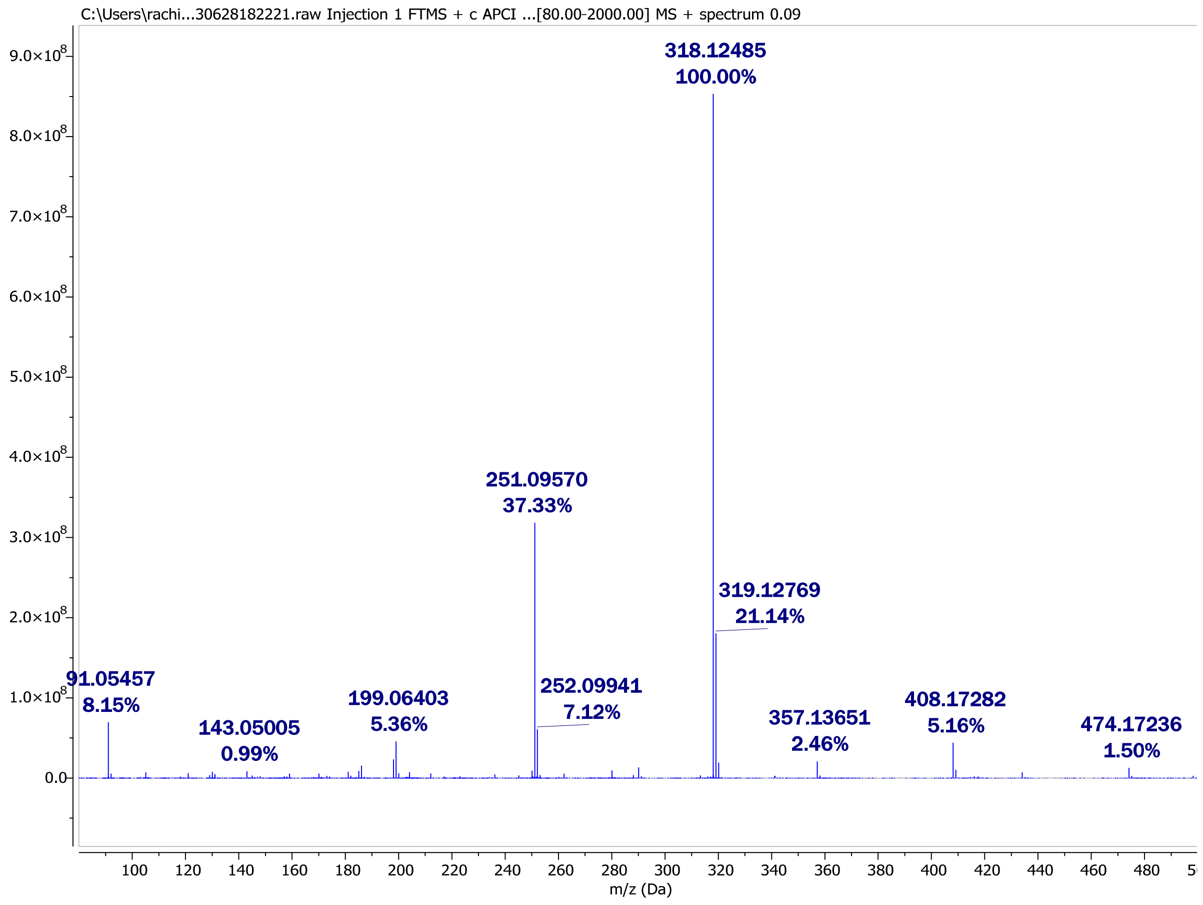

Figure S12. Mass spectrum of compound **6**.

3.4. Spectra of Compound (7a–b).

3.4.1.Spectra of (1-benzyl-1H-1,2,3-triazol-4-yl)(2-((3-(4-(trifluoromethyl)phenyl)isoxazol-5-yl)methoxy)phenyl)methanone **7a**.

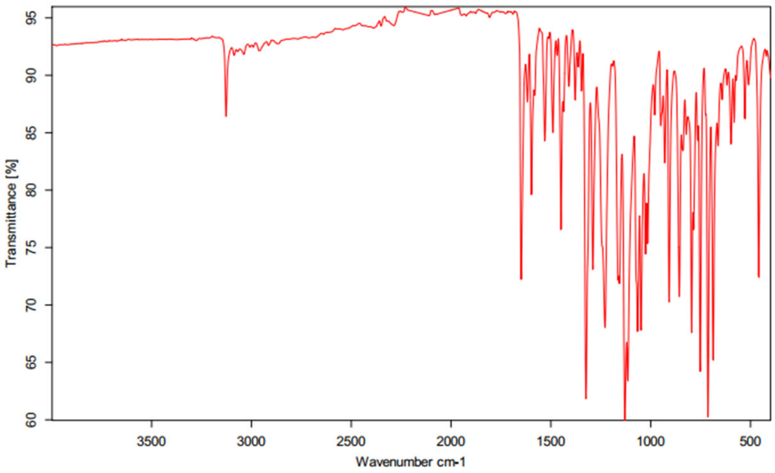

Figure S13. IR spectrum of compound **7a**.

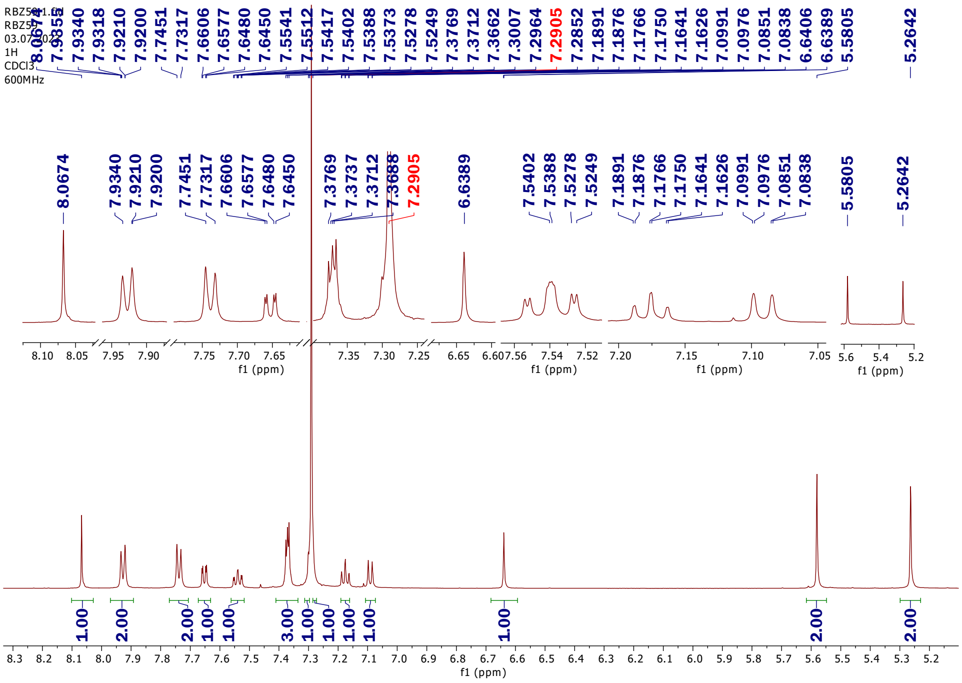

Figure S14. ^{1}H NMR spectrum (600 MHz, $CDCl_3$) of compound **7a.**

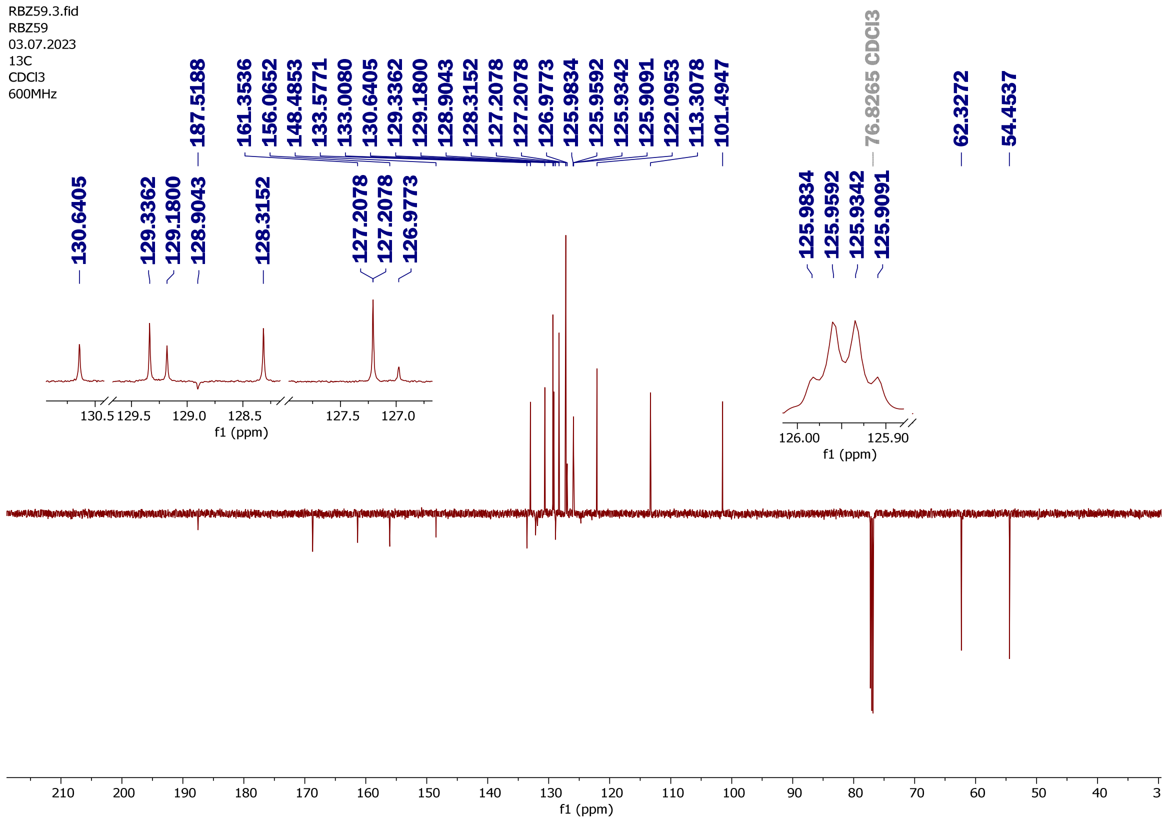

Figure S15. ^{13}C NMR spectrum (150 MHz, $CDCl_3$) of compound **7a**.

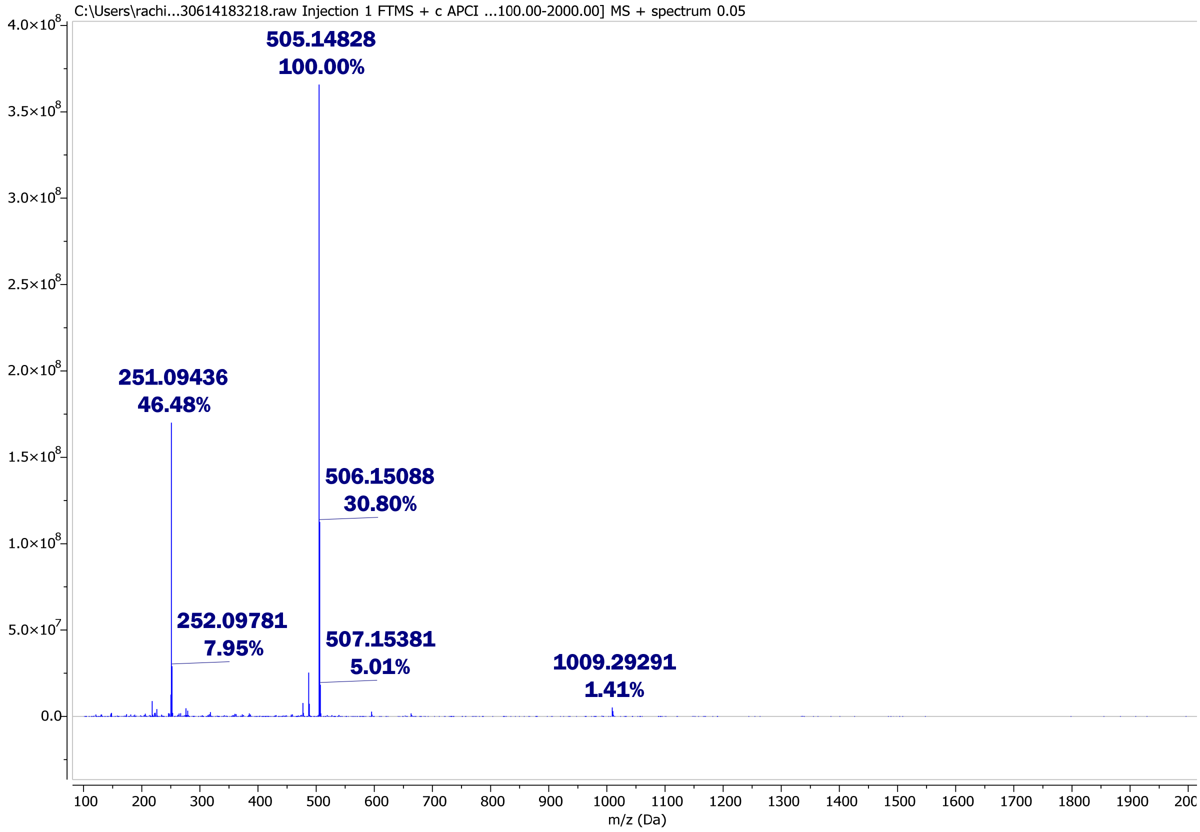

Figure S16. Mass spectrum of compound **7a**.

3.4.2. Spectra of (1-benzyl-1H-1,2,3-triazol-4-yl)(2-((3-(2-chlorophenyl)isoxazol-5-yl)methoxy)phenyl)methanone **7b**.

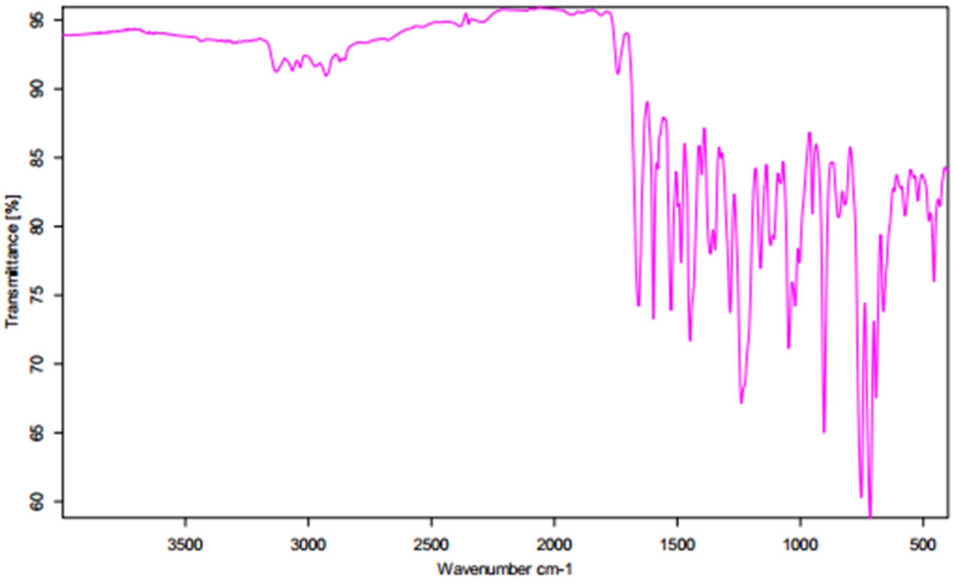

Figure S17. IR spectrum of compound **7b**.

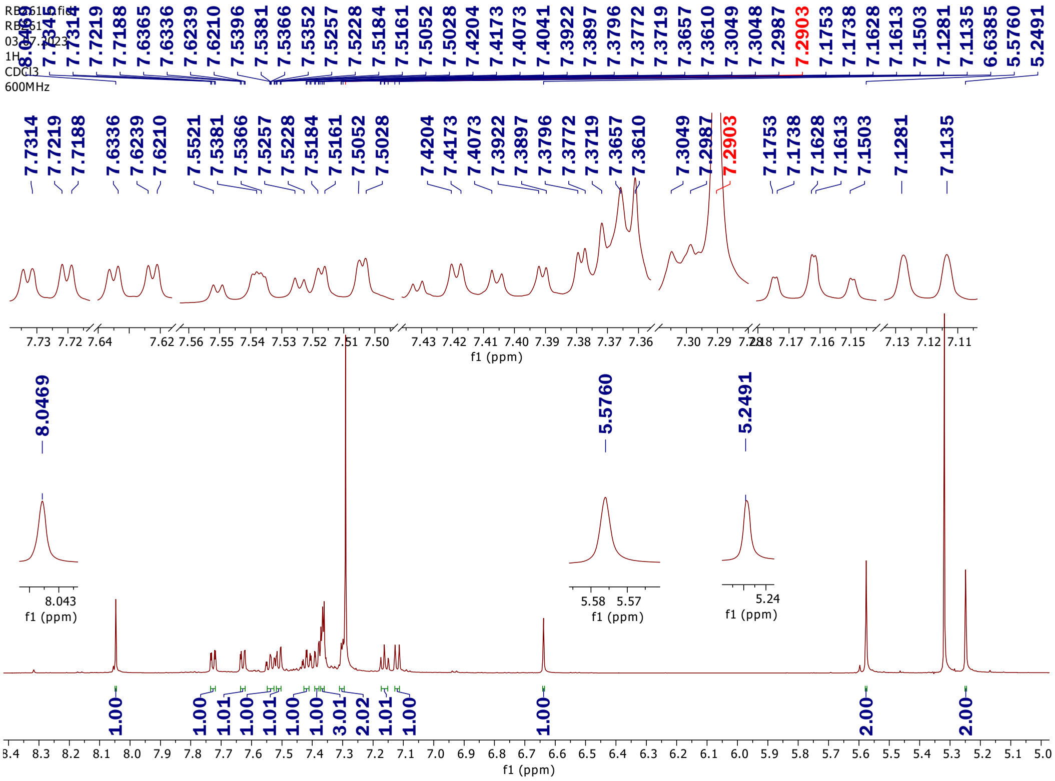

Figure S18. ^{1}H NMR spectrum (600 MHz, $CDCl_3$) of compound **7b**.

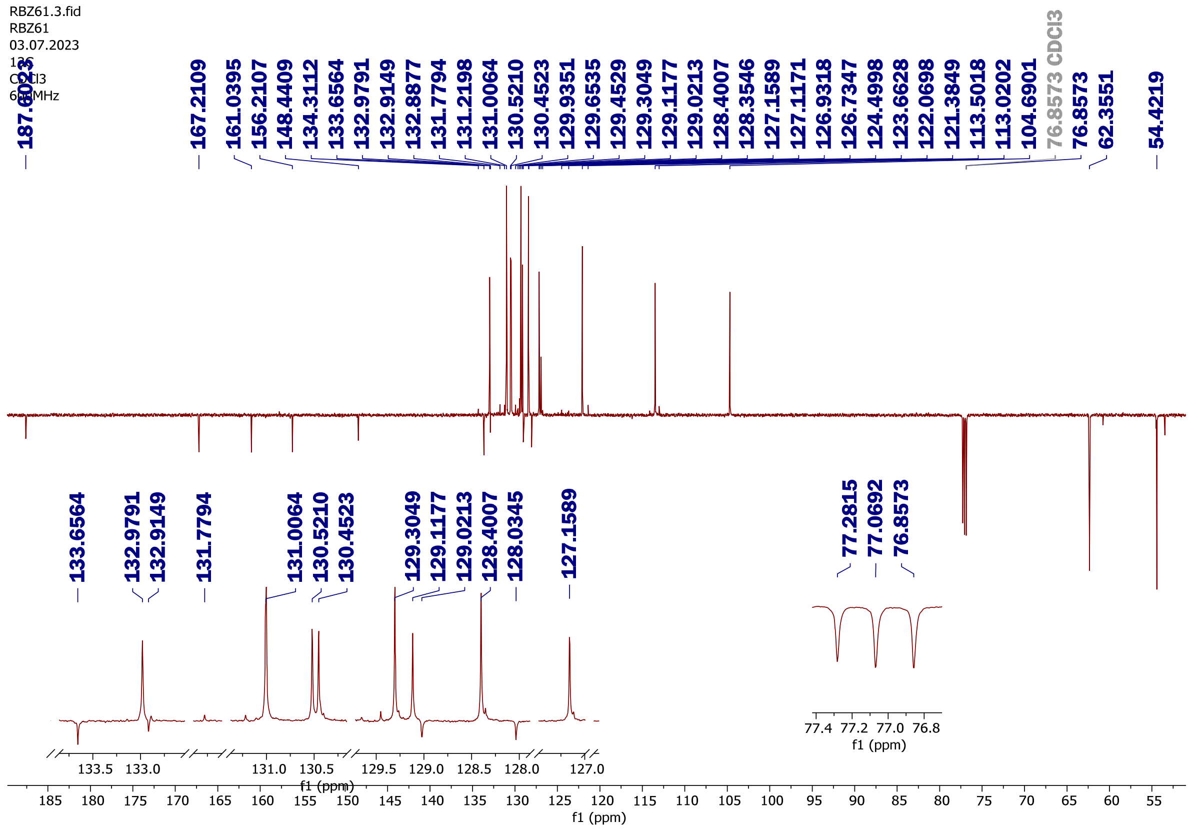

Figure S19. ^{13}C NMR spectrum (150 MHz, $CDCl_3$) of compound **7b.**

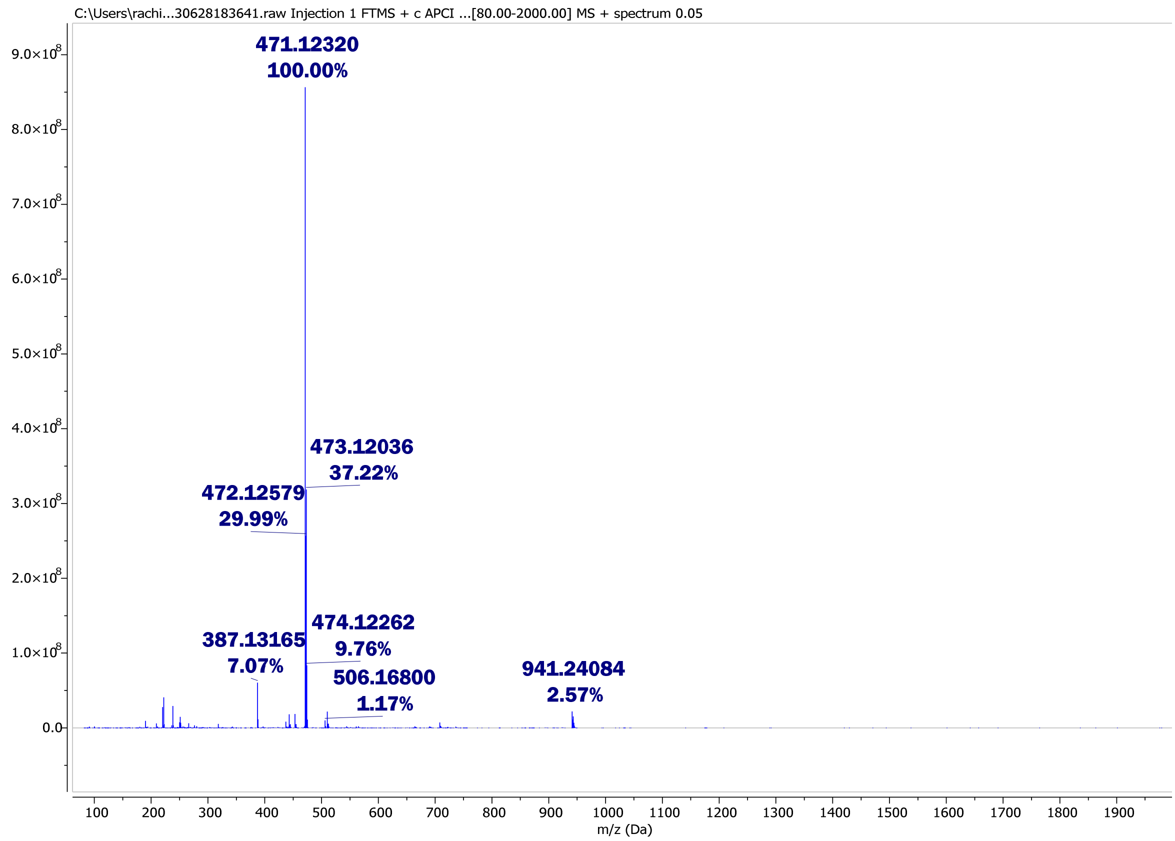

Figure S20. Mass spectrum of compound **7b**.
